# The role of oxidative stress in activity of anticancer thiosemicarbazones

**DOI:** 10.18632/oncotarget.24844

**Published:** 2018-04-03

**Authors:** Katarzyna Malarz, Anna Mrozek-Wilczkiewicz, Maciej Serda, Marta Rejmund, Jaroslaw Polanski, Robert Musiol

**Affiliations:** ^1^ Institute of Chemistry, University of Silesia in Katowice, Katowice, Poland; ^2^ Silesian Center for Education and Interdisciplinary Research, University of Silesia in Katowice, Chorzów, Poland; ^3^ A. Chełkowski Institute of Physics, University of Silesia in Katowice, Katowice, Poland

**Keywords:** thiosemicarbazones, anticancer, iron chelators, reactive oxygen species, oxidative stress

## Abstract

Thiosemicarbazones are chelators of transition metals such as iron or copper whose anticancer potency is intensively investigated. Although two compounds from this class have entered clinical trials, their precise mechanism of action is still unknown. Recent studies have suggested the mobilization of the iron ions from a cell, as well as the inhibition of ribonucleotide reductase, and the formation of reactive oxygen species. The complexity and vague nature of this mechanism not only impedes a more rational design of novel compounds, but also the further development of those that are highly active that are already in the preclinical phase. In the current work, a series of highly active thiosemicarbazones was studied for their antiproliferative activity *in vitro.* Our experiments indicate that these complexes have ionophoric properties and redox activity. They appeared to be very effective generating reactive oxygen species and deregulating the antioxidative potential of a cell. Moreover, the genes that are responsible for antioxidant capacity were considerably deregulated, which led to the induction of apoptosis and cell cycle arrest. On the other hand, good intercalating properties of the studied compounds may explain their ability to cleave DNA strands and to also poison related enzymes through the formation of reactive oxygen species. These findings may help to explain the particularly high selectivity that they have over normal cells, which generally have a stronger redox equilibrium.

## INTRODUCTION

The disruption of the cellular redox homeostasis of tumor cells appears to be an attractive and promising approach for cancer therapy. Cancer cells have increased reactive oxygen species (ROS) levels compared to normal cells. This phenomenon is associated with oncogenic transformations and glycolytic metabolic adaptations, which leads to the acceleration of metabolism [1]. Thus, the high level of ROS in tumor cells renders them more susceptible to the harmful effects of the increased oxidative stress that is induced by treatment with drugs. The aforementioned effects promote the generation of ROS and/or debilitates the antioxidant system defenses in a cell [2]. This approach may be an effective strategy to eliminate abnormal cells, including colon, pancreatic, prostate and breast cancers, which are characterized by elevated basal ROS levels [3, 4]. Importantly, in cancer cells, the overexpression of P-glycoprotein and the phenomenon of multi-drug resistance (MDR) is associated with an elevated level of ROS and a modified antioxidative capacity [5]. In particular, a high level of many antioxidant proteins plays a pivotal role in the development of multi-drug resistance [6].

The overproduction of ROS may affect the regulation of the expression of certain genes and proteins that are responsible for restoring the redox balance. Among them, the most important genes are those encoding manganese superoxide dismutase (MnSOD) and catalase (CAT). MnSOD is a mitochondrial protein that is extremely efficient in scavenging superoxide anions by converting them into hydrogen peroxide, which is further eliminated by CAT in cytosol [7, 8]. In addition, glutathione (GSH), which is the most important intracellular non-enzymatic antioxidant, plays a central role in the antioxidant system of a cell. Moreover, GSH also plays an essential role in maintaining the balance of NAD^+^/NADH, NADP^+^/NADPH and GSH/GSSG, which characterize the cellular redox state [9]. Deregulation of the endogenous antioxidant systems, including enzymes (e.g. MnSOD and CAT) and non-enzymatic antioxidant (e.g. GSH), causes many alterations that lead to the induction of oxidative stress. Elevated oxidative stress results in the oxidation of the reduced form of GSH into glutathione disulfide (GSSG) or its conjugation with endogenous and exogenous electrophiles or efflux of glutathione from the cells. All of these mechanisms lead to an overall depletion of GSH and thus result in mitochondrial dysfunction [10–12]. Generally, oxidative stress can result in detrimental cellular damage including lipid peroxidation, DNA adduct formation, protein oxidation and enzyme inactivation, which in turn can lead to cell death through cell cycle arrest or the activation of certain transcription factors [13].

Iron and copper play a crucial role in regulating many redox processes that are essential for cell homeostasis [14]. Iron is important for cellular respiration, oxygen transport, ATP generation, the synthesis of heme and DNA [15]. Similarly, copper is responsible for enzyme activity, oxygen transport and cell signaling. Those elements, due to their low electron transfer energy, are co-factors for many redox enzymes [16, 17]. This explains the higher requirement for these essential metals that is observed in rapidly growing and proliferating neoplastic cells. Organic compounds that chelate the metal ions are able to alter the metabolism and cellular signaling pathways, which may be considered to be an attractive approach in cancer treatment.

The deprivation of iron through chelation leads to the activation of various cytotoxic mechanisms in a cell [18, 19]. Thiosemicarbazones (TSC) are a well-known class of compounds with extremely potent antitumor properties, which are associated with their ability to chelate metal ions [20–23]. The exact molecular mechanism of action of TSC is still insufficiently clear. Many reports have indicated that a multifaceted mechanism of action is possible for this class of compounds [23–26]. Namely, TSC may affect the cell cycle progression by trapping Fe and depleting the iron pool in cells, which leads to cell cycle arrest in the G1/S phase and the inhibition of ribonucleotide reductase (RR), which is an enzyme that is necessary in DNA synthesis [27, 28]. Other studies have demonstrated that TSC can trigger apoptosis through the up-regulation of the metastasis suppressor protein, N-myc Downstream Regulated Gene 1 (Ndrg1) [29]. Furthermore, Ndrg1 expression is induced in response to various forms of cellular stress [30], which may be associated with the formation of redox-active metal complexes. Such complexes are known to produce reactive oxygen species *via* the Fenton reaction [23]. The latter mechanism is especially significant due to the emerging new targets in anticancer oxidative therapy, as well as its ability to increase the selective of activity of iron chelators against cancer cells [20, 23].

**Figure 1:**
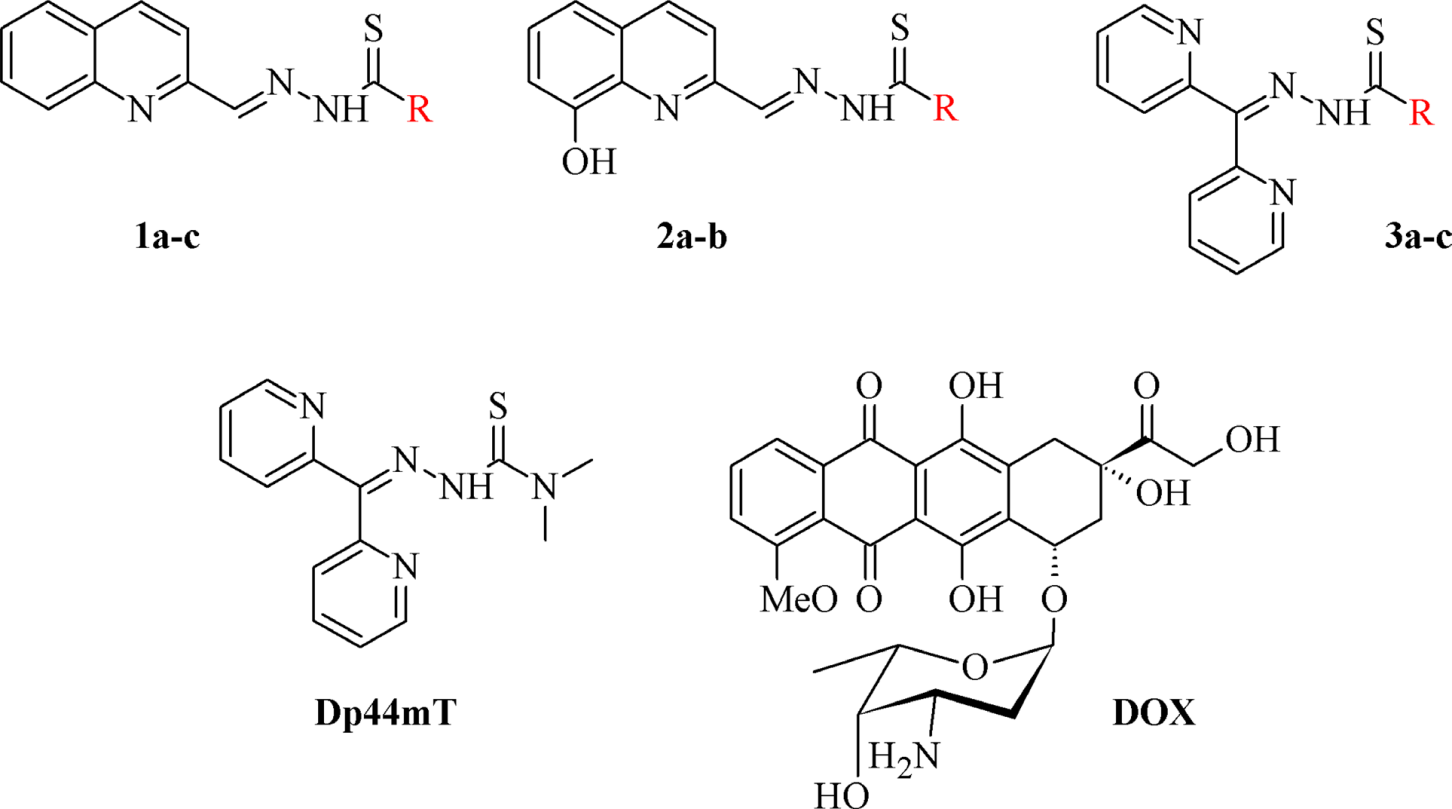
Structures of the studied compounds

Currently, this class of compounds is being extensively investigated in clinical trials. In fact, the first inhibitor of RR–Triapine has completed phase II, while DpC has recently entered phase I of clinical trials (NCT02688101) [31, 32]. Among the most active TSC are compounds that are based on the dipyridylketone scaffold such as DpC, which is an analog of a first-generation compound – Dp44mT (Figure 1). Although both compounds have exhibited a potent and selective activity against a variety of aggressive solid tumors *in vitro* and *in vivo*, DpC has also demonstrated a high tolerability *in vivo* [23, 29, 33].

The broad investigation of TSC has led to even more active compounds with a high selectivity [34, 35]. Moreover, we reported that the relationship between the structure and activity of these compounds, in particular the presence of soft donor atoms, plays a key role in the formation of redox active iron complexes [35]. More recently, other TSC derivatives have been applied as enhancers in photodynamic therapy (PDT) [36, 37]. Moreover, it has been reported that combining the copper complexes of TSC with azines eliminates cancer cells by inducing oxidative stress [38].

Although continuous efforts have allowed many aspects of the mechanisms of the activity of TSC to be clarified, other questions have been raised as the activity of the metals appears to be more complex. Iron mobilization from cytosol has also been observed to prevent the uptake of Fe from transferrin for active compounds [39]. On the other hand, it has been reported that Dp44mT and their complexes with iron and copper cause apoptosis by generating cytotoxic ROS [23, 40]. Moreover, Akladios *et al.* reported that in some TSC, the addition of metal cations may increase activity [41, 42]. This observation led to the alternative use of TSC as ionophores. In fact, the structure of the metal-TSC complexes seems to be crucial for the overall ability to chelate ions as has been revealed in Triapine [43]. Thus, more comprehensive investigations are necessary to fully understand the complex molecular mechanism of action of TSC chelators [44].

In the current study, we explored the redox potency and mechanism of action of novel TSC presented in Figure 1. We selected a series of the most effective anticancer TSC that are based on dipyridylketone and quinoline from among those that have been described recently [35, 36]. Moreover, their newly synthesized analogs and doxorubicin (DOX) were used as control. For the *in vitro* cytotoxicity tests, we used a colon carcinoma (HCT116) and a breast cancer (MCF-7) cell lines with normal human fibroblasts (NHDF) as the non-malignant control. In addition to the normal toxicity assays with iron and copper ions, the redox state in the cells was investigated. Finally, the impact of any impaired antioxidant potential on the transcription factors and cell death pathways was also examined.

## RESULTS

### Studied compounds

All of the structures of the investigated thiosemicarbazone derivatives that contain the quinoline, 8-hydroxyquinoline, and di-2-pyridylketone moieties are presented in Figure 1. The compounds used in this study were selected based on their ability of inhibit the growth of HCT116 p53^+/+^ cells in sub-nanomolar concentrations. Furthermore, we synthesized two novel TSC analogs – 1b and 1c.

### Synthesis

The heteroaromatic TSC analogs were synthesized by reacting the respective heteroaromatic ketone or carbaldehyde and thiosemicarbazide under microwave irradiation. We applied here highly efficient microwave-assisted methodology, which was previously described by our group [35]. In general, the use of microwaves improved the purity of final compounds, providing the high assays of TSC without flash chromatography.

### Cytotoxicity studies

The antiproliferative activities of all of the tested compounds against HCT116 and MCF-7 cells are presented in Table 1 as IC_50_ along with experiments with the increased metal ion concentrations.

**Table 1:** Antiproliferative activity of the studied compounds

Comp.	R	Activity - IC_50_ [nM]						
		HCT116	HCT116+ Cu^2+^	HCT116+ Fe^3+^	MCF-7	MCF-7+ Cu^2+^	MCF-7+ Fe^3+^	NHDF
1a	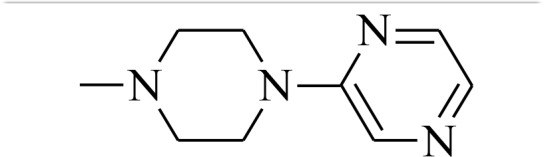	87.70 ± 43.86^a^	59.57 ± 6.45	96.91 ± 16.92	219.00 ± 15.10	44.90 ± 3.77	273.40 ± 35.65	9.44·10^3^ ± 3.84·10^3a^
1b	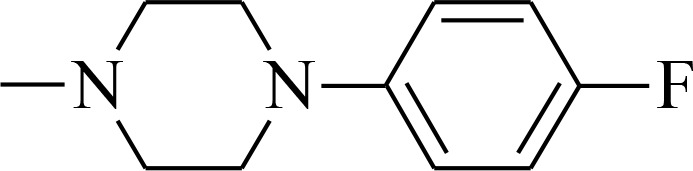	62.21 ± 8.95	19.67 ± 0.53	72.37 ± 12.90	31.39 ± 3.38	3.78 ± 0.88	24.97 ± 2.16	114.40 ± 10.30
1c	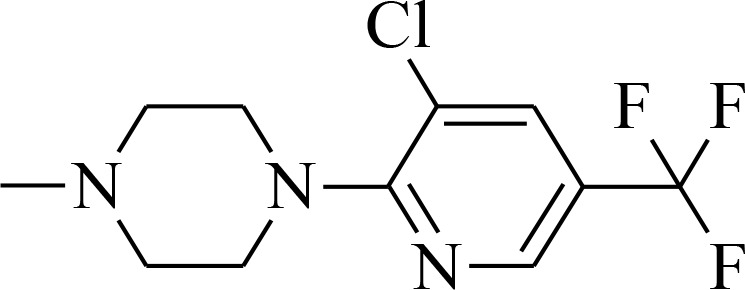	72.14 ± 9.40	7.19 ± 1.84	70.81 ± 7.28	17.25 ± 2.71	4.48 ± 1.18	39.13 ± 6.51	17.07·10^3^ ± 1.41·10^3^
2a	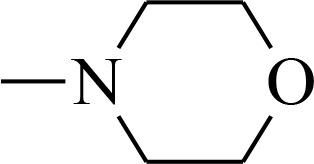	3.84 ± 1.46^a^	1.39 ± 0.44	7.57 ± 1.19	10.87 ± 2.85	2.39 ± 0.42	8.83 ± 1.29	10.64·10^3^ ± 3.48·10^3a^
2b	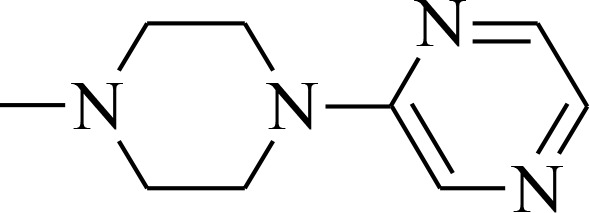	50.50 ± 28.10^a^	4.17 ± 1.12	78.16 ± 14.00	32.23 ± 6.85	5.49 ± 1.19	35.83 ± 5.82	12.14·10^3^ ± 3.58·10^3a^
3a	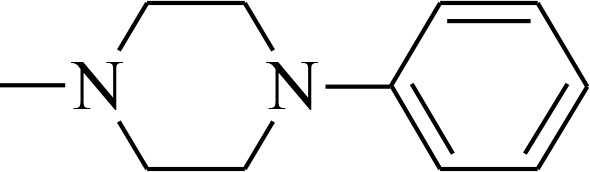	0.81 ± 0.13^a^	0.68 ± 0.15	2.07 ± 0.78	11.98 ± 2.04	1.55 ± 0.55	8.96 ± 1.81	1.73 ± 0.71^a^
3b	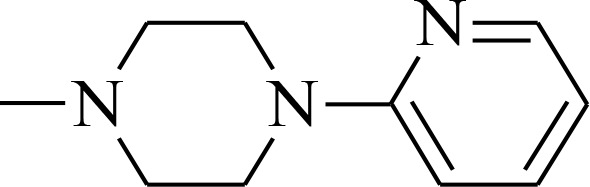	2.06 ± 0.88^a^	1.56 ± 0.44	9.94 ± 2.69	4.10 ± 0.93	0.54 ± 0.15	5.35 ± 0.66	160.00 ± 40.00^a^
3c	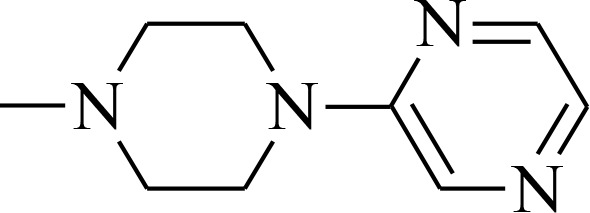	37.80 ± 3.05^a^	4.95 ± 1.64	29.53 ± 3.69	25.60 ± 6.74	4.41 ± 1.50	16.58 ± 2.25	13.85 ± 0.03^a^
Dp44mT	-	1.40 ± 0.13^a^	0.31 ± 0.06	1.77 ± 0.36	0.38 ± 0.15	0.13 ± 0.04	0.96 ± 0.32	15.38·10^3^ ± 5.06·10^3a^
DOX	-	91.00 ± 6.96	62.50 ± 8.24	84.84 ± 11.22	404.90 ± 38.00	335.60 ± 65.35	413.60 ± 59.05	137.00 ± 26.00

^a^data from [35].

Compound 3a was the most potent subnanomolar inhibitor in the colon cancer line followed by 3b and 2a, that were roughly as active as Dp44mT. However, 3a was also the most toxic against normal fibroblasts (NHDF). In terms of selectivity, compounds 1a, 2a and 2b appeared to be the most useful with 2a having a selectivity index (for HCT116) equal to 2.8·10^3^ (Supplementary Table 1). Interestingly, all of the compounds that were tested showed various patterns of activity against the MCF-7 cell line. 3b was the most active while 1c had the highest selectivity (1.0·10^3^ - for MCF-7).

The influence of iron on the toxicity of TSC is particularly interesting. We found that the addition of Fe^2+^ ions completely eliminated the activity of all of the compounds that were tested within the concentration levels (data not presented). On the other hand, in the presence of Fe^3+^, the TSC retained their activity against HCT116 and MCF-7 cells. At first glance, these results may seem in contrast to the literature data where Triapine and its derivatives were considerably less active when administered with Fe^3+^ [43]. This, however, may suggest how important the basal level of iron and ROS in cancerous cells is. We also examined the influence of copper ions on the cytotoxic effects that are induced by TSC in HCT116 and MCF-7 cells. As expected, we observed that supplementation with this redox-active metal caused a significant increase in the cytotoxicity of the TSC. In general, we did not observe any uniform patterns of improved cytotoxicity with copper for the tested TSC in the HCT116 and MCF-7 cell lines. Nevertheless, similar to the above, compounds containing the di-2-pyridylketone moieties (3a, 3b and Dp44mT) and the 8-hydroxyquinoline derivative (2a) exhibited the highest Cu^2+^-dependent cytotoxicity against both cancer cell lines. On the other hand, after interacting with Cu^2+^, we observed the greatest improvement in cytotoxicity in the case of the 1c and 2b compounds in HCT116 cells (more than a ten-fold increase) and for 1b, 3a, 3b (eight-fold) in MCF-7 cells.

### Reactive oxygen species formation – microscopic images

**Figure 2:**
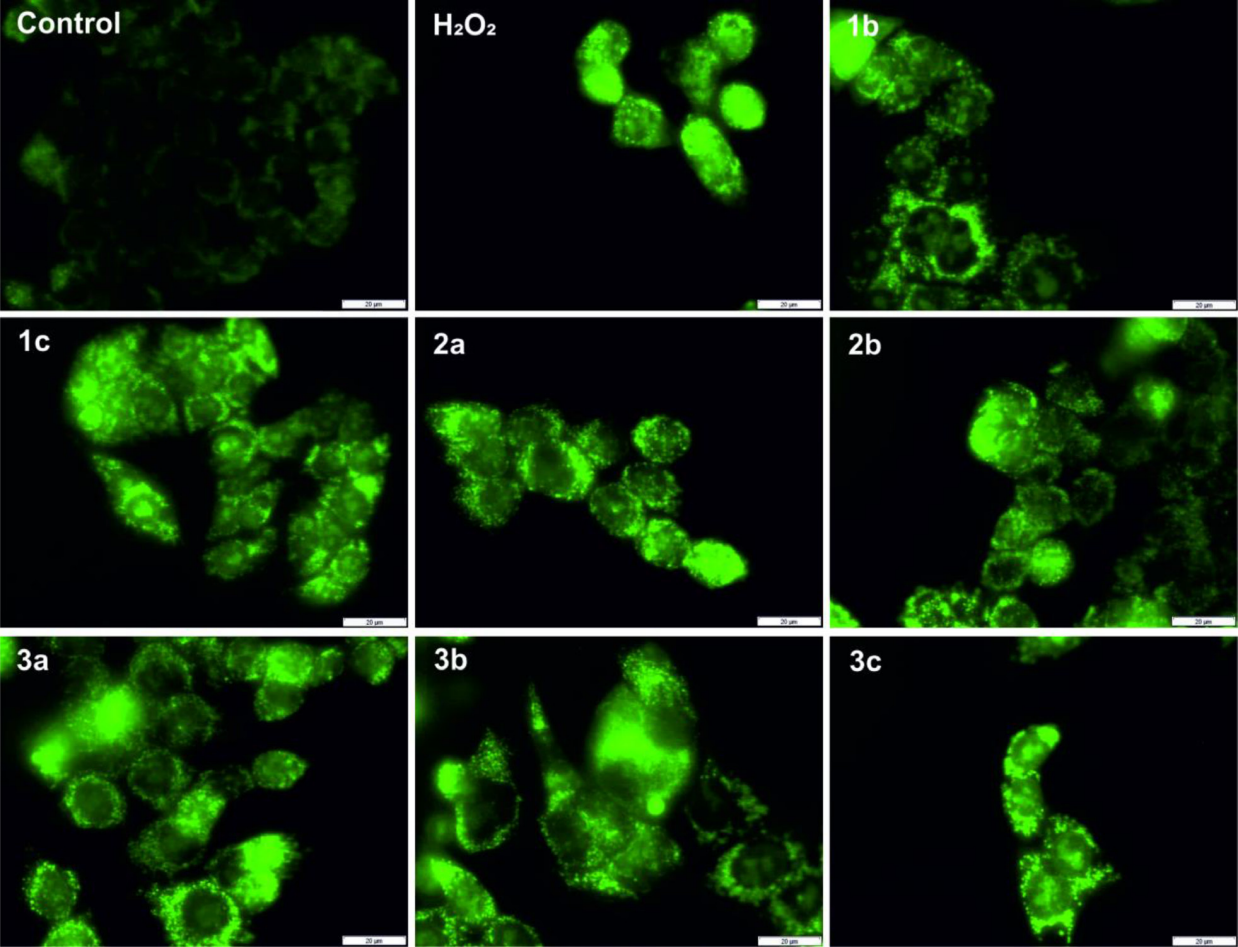
Formation of reactive oxygen species in HCT116 cells after 24 h treatment with the tested compounds (concentration 2 × IC_50_) and a 15-min incubation with hydrogen peroxide (100 µM), which was used as the positive control The negative control consisted of untreated cells. Scale bars = 20 μm.

To confirm the increased redox activity of the complexes with the tested compounds, we performed fluorescence imaging using CellROX, which binds to DNA upon oxidation and thus, its signal is localized primarily in the nucleus and mitochondria. The results are presented in Figure 2.

Interacting with transition metal ions may increase the ROS level in the cytosol and cause cell death. This mechanism has also been suggested for TSC [21]. All of the thiosemicarbazones that were tested increased the level of ROS significantly and were similar to hydrogen peroxide, which was used as the positive control. Additionally, after treatment with the tested compounds, we observed a strong fluorescence signal, which may primarily be focused on the mitochondria and nucleus.

### Time dependent measurement of ROS level

**Figure 3:**
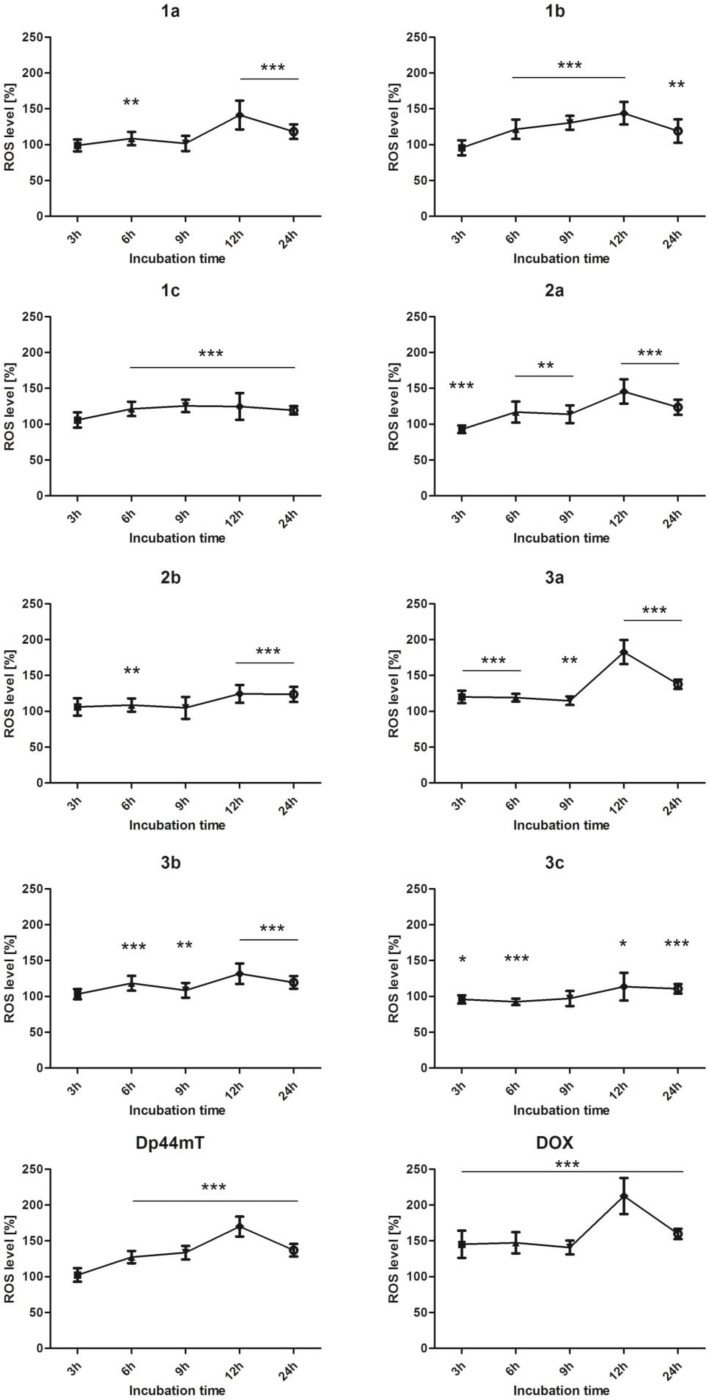
Effect of the tested compounds (1 µM) and DOX (5 µM) on the level of ROS in HCT116 cells Data normalized to untreated cells (control). Results are shown as the mean ± SD of three independent measurements. Data were analyzed using the Student's *t*-test: ^*^p < 0.05, ^**^p < 0.01, ^***^p < 0.001 compared to the control group.

As presented in Figure 3, the ROS level gradually rose over the time and reached the highest level after twelve hours of incubation, after which it dropped rapidly. Although this pattern was observable in all of the tested compounds, it appeared to correlate well with the activity of the TSC. Compound 3a had the strongest effect, followed by Dp44mT and 2a. On the other hand, 1a-c and 3c caused only a minute increase in the ROS level. This may confirm that the main aspect of the multiway mechanism of the activity TSC relies on the Fenton and Haber-Weiss reactions.

**Scheme 1:**
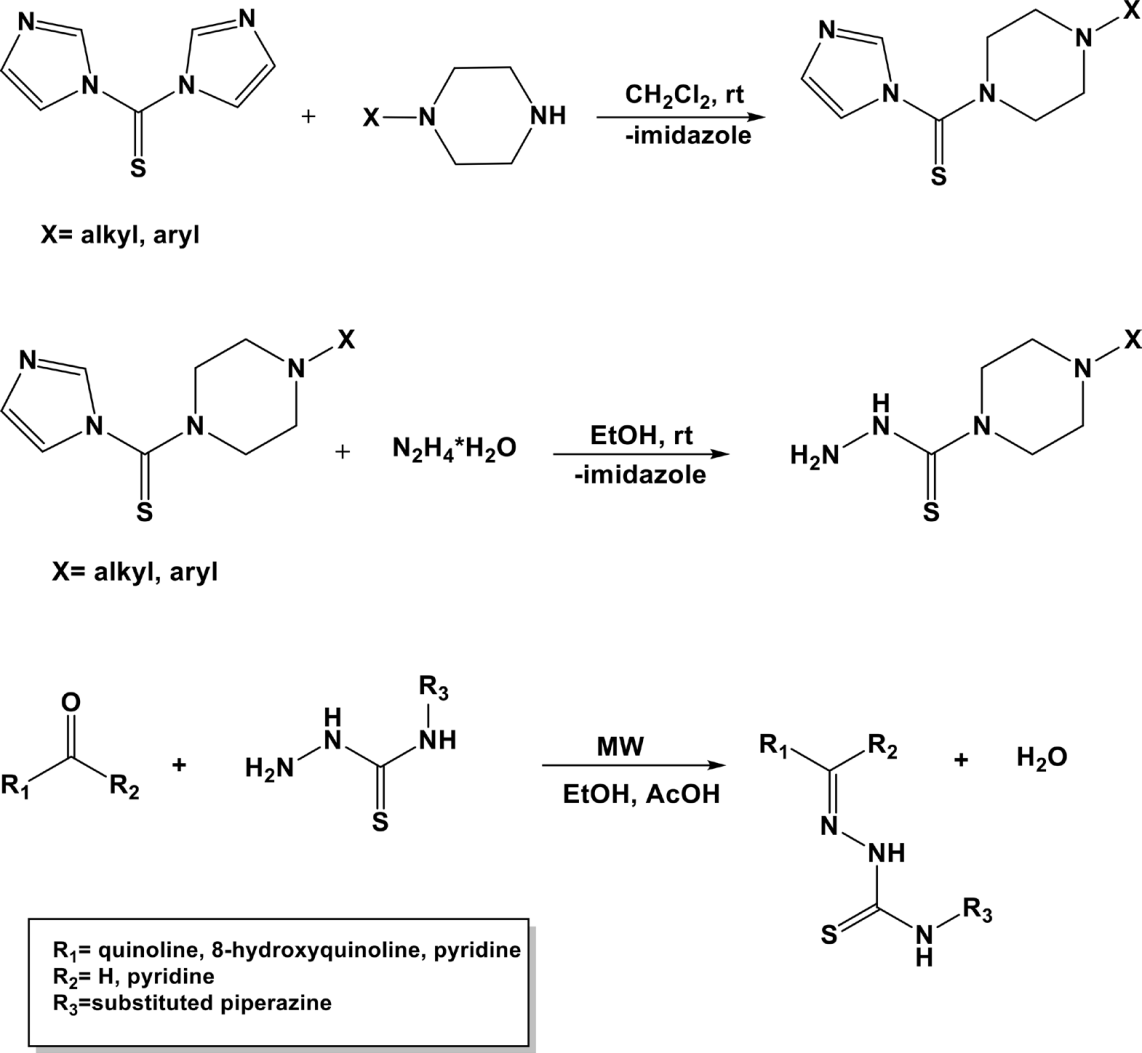
General method for synthesis of studied TSC.

### Time dependent measurement of glutathione level

**Figure 4:**
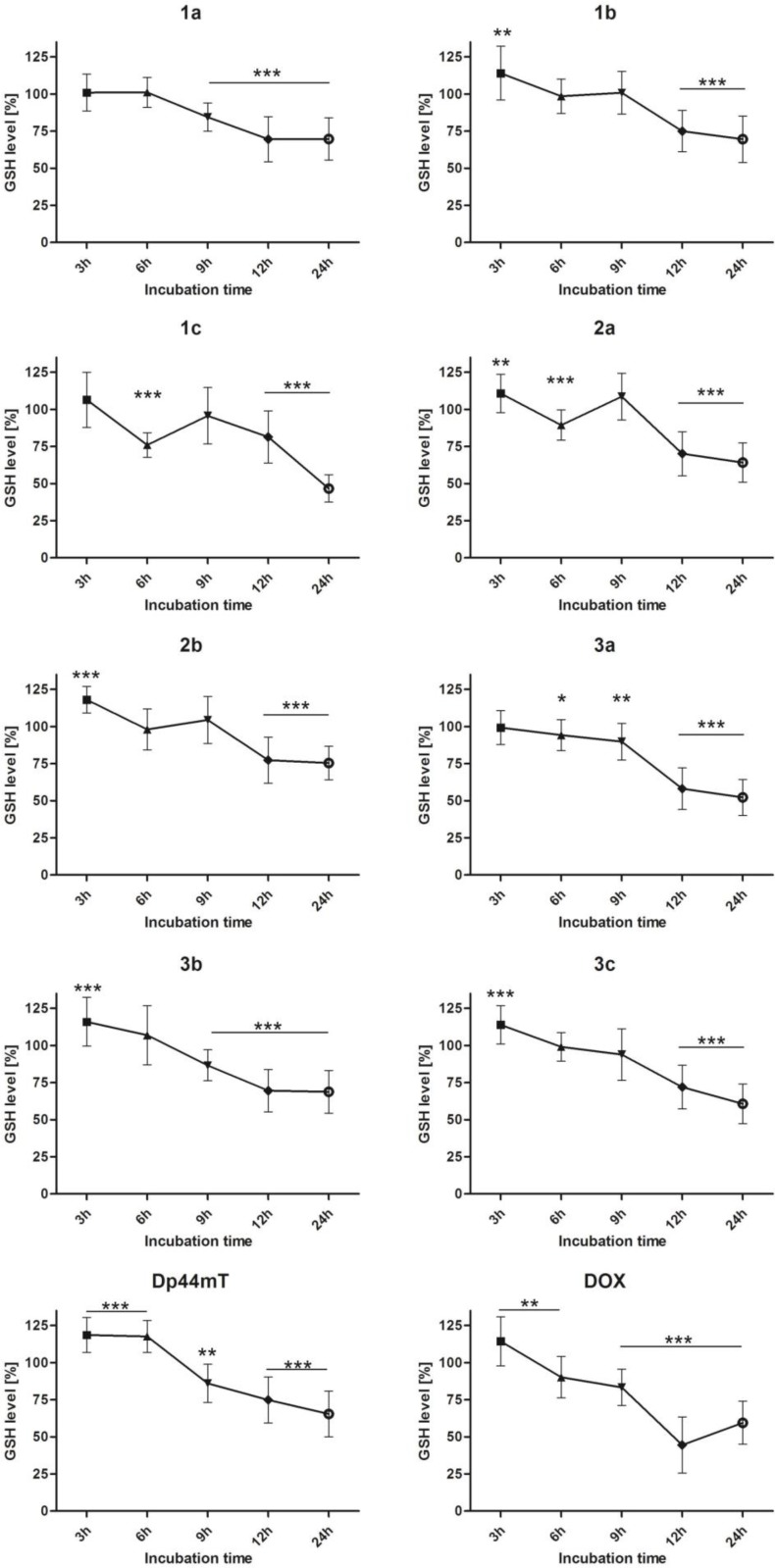
Effect of the tested compounds (1 µM) and DOX (5 µM) on the intracellular GSH content of HCT116 cells Data normalized to the untreated cells (control). Results are shown as the mean ± SD of three independent measurements. Data were analyzed using the Student's *t*-test: ^*^p < 0.05, ^**^p < 0.01, ^***^p < 0.001 compared to the control group.

Previous results prompted us to study the influence of the tested TSC on the GSH level. We performed experiments in the same conditions (as for the ROS level), but this time the concentration of the natural antioxidant (GSH) was measured. Results are shown in Figure 4.

A high level of ROS led to the oxidation of glutathione and diminished the overall antioxidative potential in a cell. Time-dependent changes in the GSH level confirmed the increased vulnerability of the cells to ROS-dependent damage. This in turn prompted us to evaluate the gene expression under the oxidative stress that was induced by the TSC.

### The antioxidative genes expression

**Figure 5:**
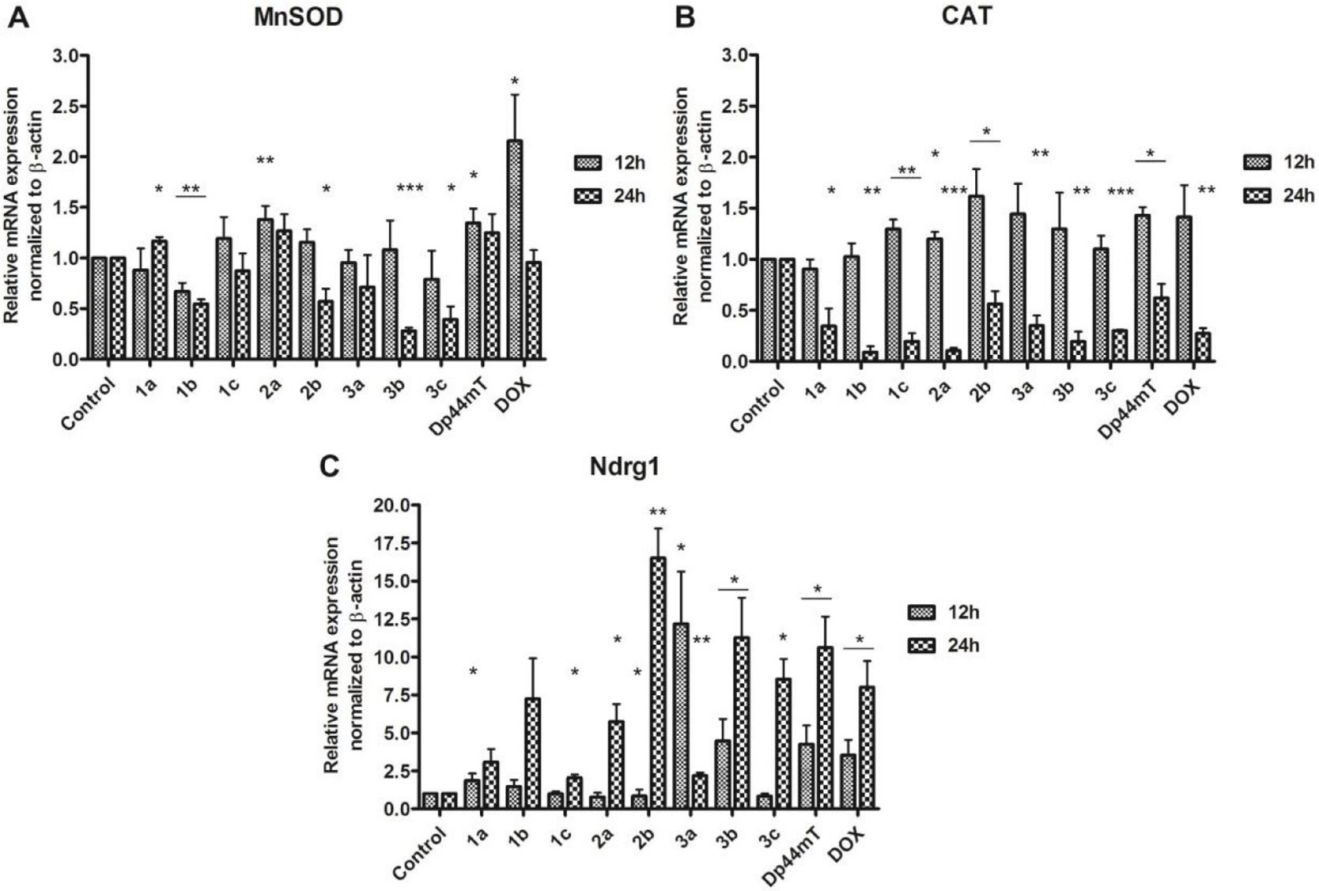
mRNA expression of MnSOD, CAT and Ndrg1 in the HCT116 cell line after incubation with TSC (1 µM) and doxorubicin (5 µM) Results are shown as the mean ± SD of three independent measurements, each in triplicate. Data were analyzed using the Student's *t*-test: ^*^p < 0.05, ^**^p < 0.01, ^***^p < 0.001 compared to the control (untreated cells).

The antioxidative genes concentration levels are presented in Figure 5.

In general, most of the tested TSC caused a decrease in the expression of MnSOD (Figure 5) progressing with incubation time, except of 2a, where slight increase can be noticed. For compound 1a however, after the initial decline (for 12 h) there is a small increase in expression of MnSOD. In the case of catalase, the effect was strongly dependent on time as its level was higher for the active compounds (2b, 3a, 3b, 1c) after the first 12 h. Then, after the next 12 h, it decreased dramatically for all of the thiosemicarbazones that were tested. This downregulation of MnSOD and CAT could be explained by the interruption in the cellular oxidative equilibrium towards the induction of strong oxidative stress. The level of ROS is so high, that mechanisms responsible for protecting the cell collapsed. Therefore, leading to trigger the pathways involved in the cell death. One of the factors engaged in the apoptosis induction is Ndrg1 protein. As is shown in the Figure 5 the Ndrg1 gene level rose considerably after 24 h of incubation with the TSC. This effect was the strongest for 2b, 3b, and 3c. We also observed significant increase for 3a, but after 12 h. These results indicate that the cell entered on the apoptosis pathway [45].

### Western blot analysis of the cell cycle and apoptosis proteins

**Figure 6:**
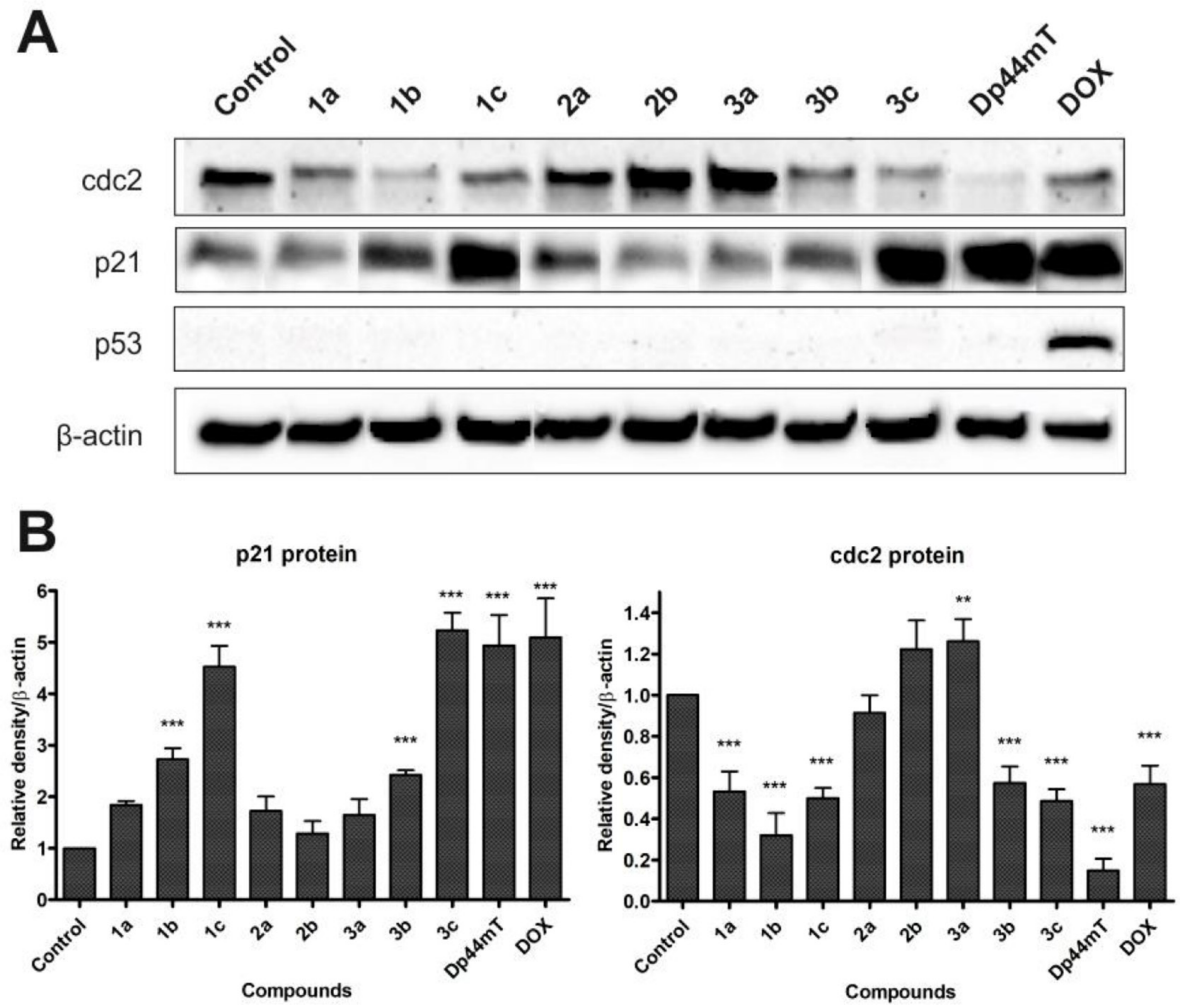
Influence of the tested compounds (1 µM) and DOX (5 µM) on the expression of cdc2, p21, p53 and β-actin in HCT116 cells (**A**). Data for p21 and cdc2 were analyzed using one-way ANOVA with Bonferroni’s post-hoc test: ^*^p < 0.05, ^**^p < 0.01, ^***^p < 0.001 compared to the control (**B**).

Antioxidant genes expression indicated that tested TSC influenced the ROS generation in cell. This prompted us to carry out further studies designed to reveal proteins responsible for cell cycle and death. As is shown on Figure 6 we explored p21, p53, and cdc2 proteins. We observed no influence on the p53 among all of the tested compounds, except DOX. Densitometric analysis of proteins indicated a marked up-regulation of p21 after incubation with 1c, 3c and Dp44mT (Figure 6B). For compounds 1b, and 3b we also noticed the increased p21 level, but in a lesser degree. In the case of cdc2 we detected a significant growth just for 3a. For the rest of the tested compounds we register down-regulation, which takes the lowest values for Dp44mT and 1b.

### Cell cycle assay

**Figure 7:**
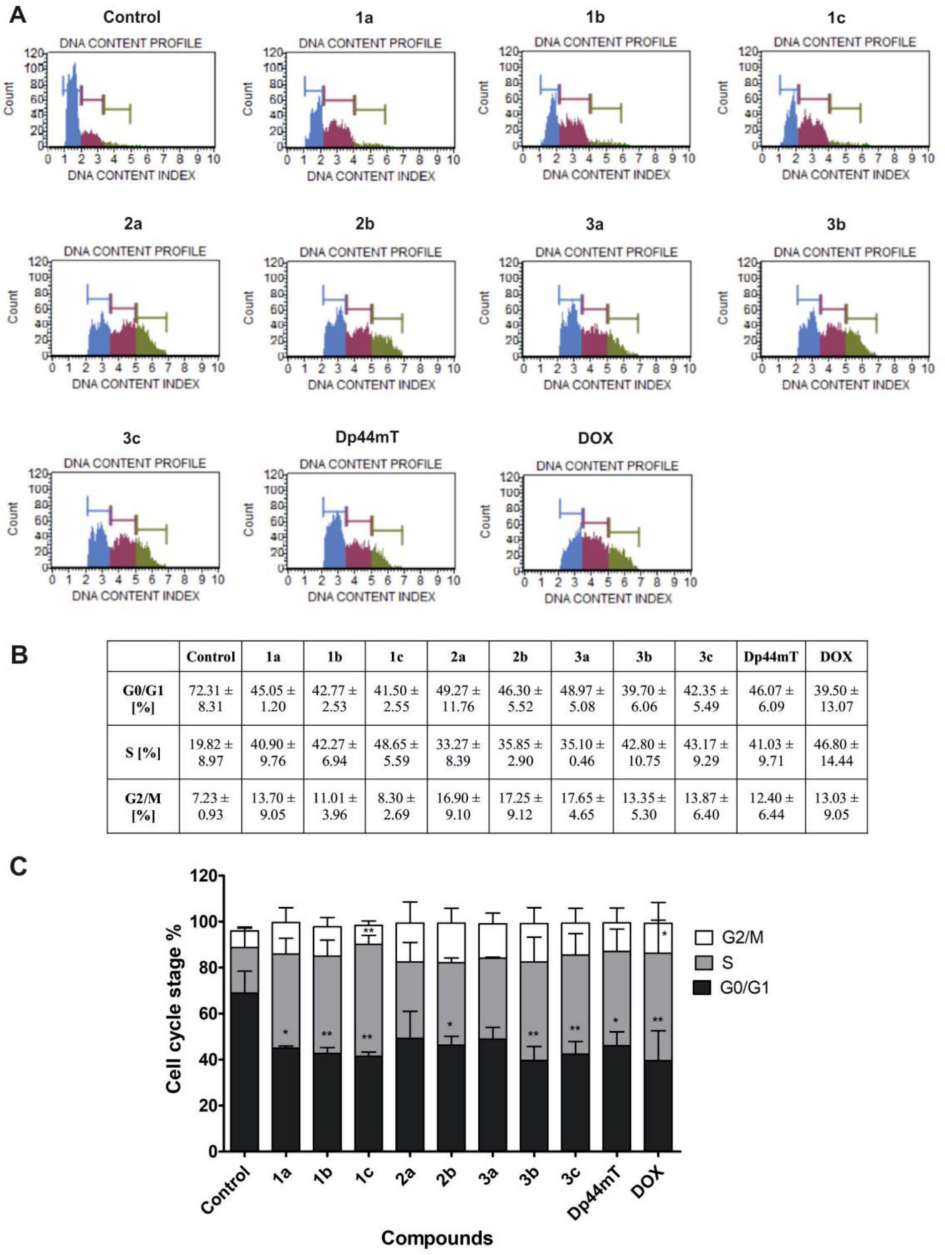
Impact of TSC (1 µM) and DOX (5 µM) treatment on the regulation of the cell cycle in HCT116 cells The histograms show the distribution of cells in the G0/G1, S and G2/M phases of the cell cycle for one of the experiments (**A**). The table shows the mean ± SD percentage of cells in the G0/G1, S and G2/M phases of the cell cycle from three independent experiments (**B**). Data were analyzed using one-way ANOVA with Bonferroni’s post-hoc test: ^*^p < 0.05, ^**^p < 0.01, ^***^p < 0.001 compared to the control (**C**).

We examined the effect of the tested TSC and DOX on the regulation of the cell cycle in HCT116 cells after 48 h of incubation (Figure 7). Generally, we observed a significant decrease in the percentage of cells in the G0/G1 phase after treatment with all of the tested compounds. Specifically, one of the most active compounds – 3b diminished the cell count in the G0/G1 phase to 39% compared to the untreated cells (72%) (Figure 7B). Additionally, all of the TSC caused a significant increase of the fraction cells in the S phase of the cell cycle. In general, some differences after treatment with the compounds in series 1, 2 and 3 were observed. The compounds of 1a-c and 3b-c showed a significant increase in this proportion of cells of 40–48%. These results were comparable to those of Dp44mT (41%), while in the case of 2a, 2b and 3a, smaller effect was observed (33–35%). Thus, all of these data suggest that TSC may also induce cell death through the cell cycle arrest in the S and G2/M phases. For example, the compounds in series 2 and compound 3a caused an increase in the cell count in the G2/M phase to 17% compared to the untreated cells (7%) (Figure 7B).

### Annexin V binding assay

**Figure 8:**
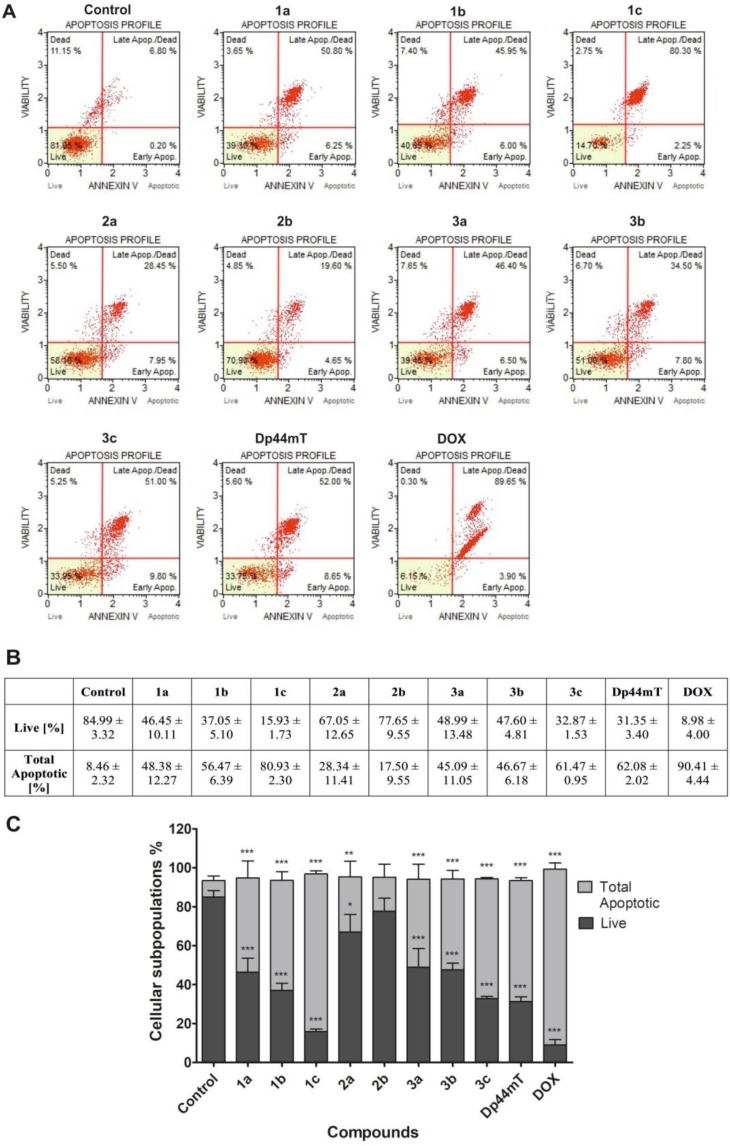
Evaluation of apoptosis induction in HCT116 cells after 48 h of treatment with TSC (1 µM) and DOX (5 µM) The histograms show the percentage of early and late apoptosis for one of experiments (**A**). The table shows the mean ± SD percentage of live, early and late apoptotic cells from three independent experiments (**B**). Data were analyzed using one-way ANOVA with Bonferroni’s post-hoc test: ^*^p < 0.05, ^**^p < 0.01, ^***^p < 0.001 compared to the control (**C**).

The evaluation of apoptosis in the HCT116 cell line was confirmed using Annexin V staining. Results are presented in Figure 8.

As is presented in Figure 8, a significantly increased population of total apoptotic cells after treatment with tested the compounds can be observed. Moreover, the strongest effect was observed for 1c, 3c, Dp44mT and DOX, which correlates with their ability to significantly up-regulate the p21 protein. In the case of the 2a-b compounds, the lowest proportion of apoptotic cells was observed, which may be associated with a late entry into apoptosis or undergoing a mitotic catastrophe. In this case, a higher chelation ability and the disruption of the antioxidant potential of a cell may be connected with a stronger obstructive effect on cell cycle progression and a lower overall apoptotic cell count.

### Intercalation

**Figure 9:**
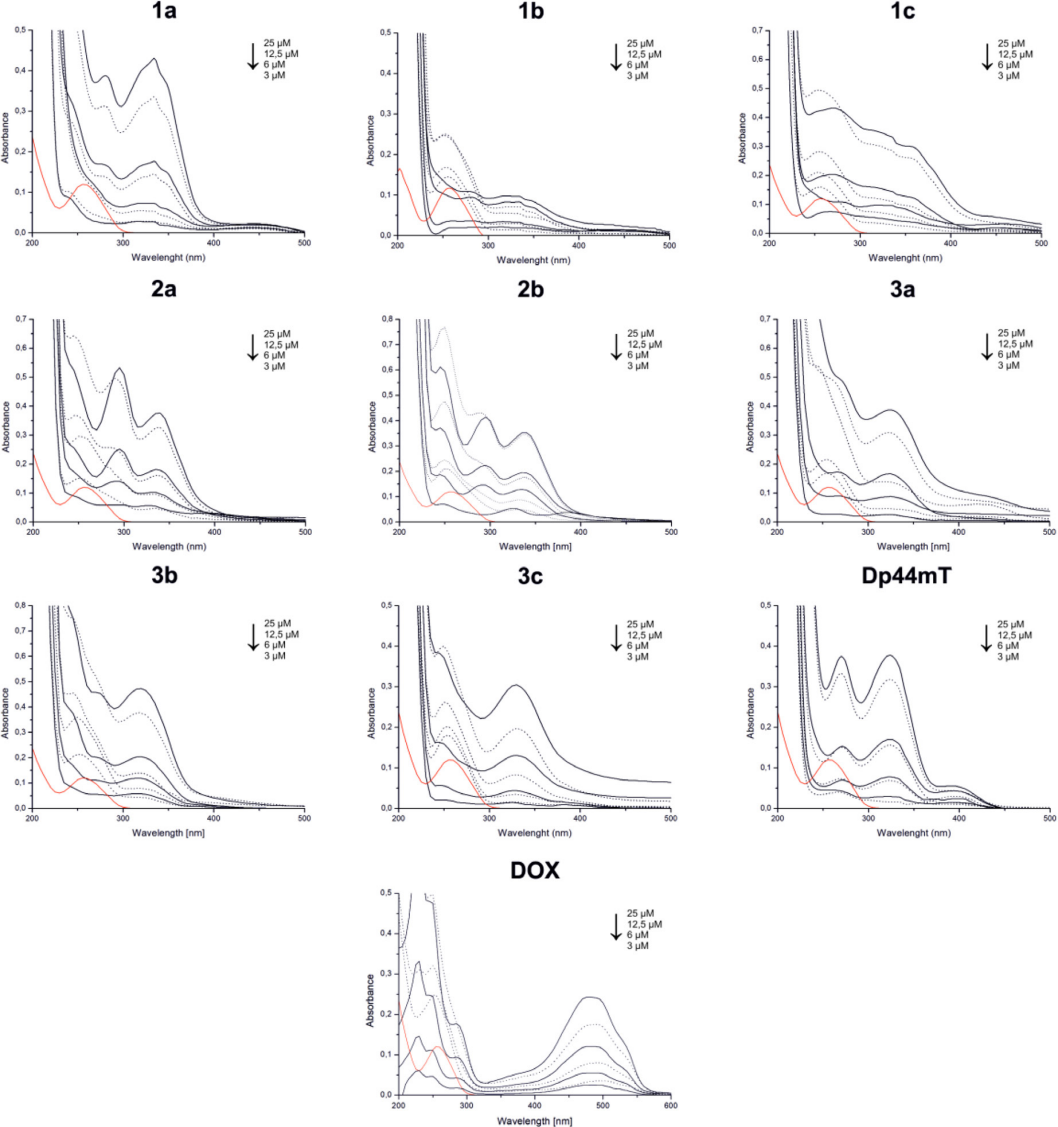
Absorption spectra of the tested thiosemicarbazones and DOX in the absence of CT-DNA (solid line) and in the presence of the CT-DNA (dotted line) in phosphate-buffered saline Red line indicates the CT-DNA alone in PBS.

Further, we studied the ability of tested thiosemicarbazone derivatives to bind DNA strains. The absorption spectra of the tested compounds in the absence and presence of calf-thymus DNA, which is presented in Figure 9 and Table 2, summarize the absorption spectral properties of the tested thiosemicarbazones. Our results indicate that DNA intercalation is strongly possible in the tested TSC, which results in a decrease in band intensities and a small shift of the wavelength. These changes in the band intensities of the spectra (hypochromism) are caused by the contraction of the DNA helix axes and also from the conformational changes on the molecules of DNA [46]. TSC (3a-c) with a fragment of dipyridyl ketone revealed the strongest hypochromism of about 28.2%, 31.9% and 28.5%, respectively. These values are similar to the reference – doxorubicin, which is a well-characterized DNA-intercalation drug [47]. In our experiments, we observed a significant decrease in the absorption intensity of about 34.2% for doxorubicin. Interestingly, Dp44mT showed a very weak ability to intercalate DNA, which has also been confirmed by other groups [23, 48]. However, thiosemicarbazones 1a-c, which are based on the quinoline moiety, showed good intercalation properties and strong hypochromism of about 22.0%, 24.2% and 22.6%, respectively, was observed.

**Table 2:** Absorption spectral properties of the tested compounds bound to CT-DNA

Compound	Absorption λ_max_ [nm]	Changes in absorbance	% hypochromism	Δε M^−1^ cm^−1^	blue/red shift^*^ [nm]
1a	282; 334	hypochromism	22.0	3175.6	1
1b	324	hypochromism	24.2	2313.3	10
1c	272; 310	hypochromism	22.6	2695.6	4
2a	295; 330	hypochromism	15.7	1935.6	10
2b	295; 338	hypochromism	9.4	1888.9	5
3a	325	hypochromism	28.2	4120.0	5
3b	320	hypochromism	31.9	6133.3	5
3c	330	hypochromism	28.5	3220.0	2
Dp44mT	272; 324	hypochromism	8.2	1673.3	2
DOX^a^	480	hypochromism	34.2	3235.6	10

^*^for the wavelengths of the maximum absorption for compounds alone and the DNA-bound compounds. ^a^data from [49].

## DISCUSSION

Dipyridyl- and quinolinyl-based TSC are among the most potent antiproliferative compounds of this class [23]. Among them, we recently identified a series of derivatives that appeared to be thousands of times more effective than Triapine against several human cancer cell lines [35]. In this paper we present several compounds, which possess strong anticancer activity (1a, 2b, 3a, 3b), and a promising selectivity index. Antiproliferative activity of these compounds was investigated along with apparent mechanism of action. In general TSC can be regarded as metal chelators. According to the hypothesis presented by Ding and Lind metal chelating compound can be classified as chelator, metal shuttle or ionophore regarding to the intrinsic mechanism of action [50]. This can be distinguished by testing the activity changes in metal overload or complexes. For TSC however series of complexes were synthesized and tested providing ambiguous results [51]. Both the increase [21] and decrease [23] of activity have been reported for various complexes. For active compounds, that are tested in much smaller concentrations than metal ions, the ionophoric mode (ligand transports multiple ions through membranes) seems to be predisposed [40, 52, 53]. To test the ionophore hypothesis, we studied the influence of metal ions on the cytotoxicity of the TSC. Human colon cells and breast cancers are particularly suitable for this study because of their higher sensitivity to an increased ROS level due to their higher basal level [54]. Both of the cell lines (HCT116 and MCF-7) used in this study are also known for their the relatively high level of iron and iron-regulatory proteins [55–57]. Moreover, drugs that impair the level of cellular iron were found to be less vulnerable to resistance in these cells [58, 59]. All of the TSC that were tested are potent iron chelators that are able to change the iron concentration within a cell [34, 35]. The overall cytotoxic effect, however, depends on the cells susceptibility to changes in the iron and ROS levels. Similar differences have been described for the leukemia [60], neuroepithelioma [23] and neuroblastoma [61] cell lines. In the case of copper ions we observed even higher increase of the activity. One explanation for these observations may be the different chelating ability of tested TSC, as we suggested in the case of iron. Moreover cancer cells are more sensitive for the change of the Cu^2+^ level as basal level of this metal is elevated, particularly in prostate and breast cancers [62]. Copper chelators with ionophoric activity are known for their promising potency and selectivity towards cancer cells. An example of such compounds studied in clinical trials is clioquinol or bisthiosemicarbazone [16]. However the exact mechanism of their activity is not clarified, they tend to increase oxidative processes or displacing other metals from their complexes with specific proteins. The redox-active complexes of Dp44mT with lysosomes specific accumulation were reported by Lovejoy *et al.* [40]. Both of the structural fragments (quinoline/di-pyridine and thiosemicarbazone moieties) that are exploited in those new TSC are known to chelate metal ions. Considering this, we decided to investigate the mechanisms of oxidative stress induction after treatment with those highly potent TSC more deeply.

Our recent studies confirmed that TSC derivatives may be accumulated also in mitochondria [37]. This suggests that the ROS that were generated by the TSC may influence the antioxidant capacity and contribute to the disruption of mitochondria, which then triggers apoptosis. To evaluate this hypothesis, we measured the intracellular level of ROS and glutathione in time dependence manner. Results shown reverse dependence of changing ROS and GSH level. In general, ROS concentration increased along incubation time, reaching the maximum after 12 h. The opposite effect was observed for GSH. Its level decreased during the whole experiment lasting 24 h. GSH depletion is a strategy to overcome MDR-resistance and it is also an early event in cell death in response to different stimuli [12]. This, however, may occur in two different ways depending on the contribution of p53 [63, 64]. For 3a we detected the highest ROS level (12 h), among all of the tested compounds. This can be correlated with anticancer activity and confirmed hypothesis, that mechanism of action is connected with ROS generation. Therefore the next step was to measure the expression of genes responsible for maintaining oxidative balance in cell. Genes expression dynamic correlates with the antiproliferative activity of the tested compounds. Such a reduction in antioxidant proteins following ROS-induced damage is a known trigger of apoptosis [65]. The inhibition of manganese superoxide dismutase and catalase expression appears to play a crucial role in breaking down the cellular defense against increased ROS level. Additionally catalase may affect the mechanism of multidrug resistance and protect tumor cells against the induction of apoptosis [66, 67]. Ndrg1 expression is another important factor in the stress response that was observed to be considerably elevated. This gene is down-regulated in various cancers, which is often connected with p53 level [68]. Amplification of this gene is frequently reported in response to chemotherapy and leads to the inhibition of growth and metastasis. Alterations of the intracellular level of iron through chelation and facilitated mobility have been identified as one of the triggers of Ndrg1 up-regulation [22].

With this in mind, we determined the influence of the tested TSC on the expression of p53 and p21 proteins, which are involved in cell cycle progression and apoptosis induction. Interestingly, none of the tested TSC induced the expression of the p53 protein. On the other hand, western blot analysis revealed a considerable up-regulation of the p21 protein after treatment with all of the compounds that were tested. The strongest effect, comparable to DOX, among the TSC was observed for 1c, 3c and Dp44mT. Those results indicate a p53-independent pathway of apoptosis. The Ndrg1 gene has also been reported to up-regulate p21 and activate WAF1/CIP1 expression as apoptotic triggers [69–71].

Cellular ROS level is strictly related to the cell cycle. In general a concentration of oxygen radical is steadily growing from G1 through S phase to G2 + M and then drops back in quiescent state. Both antioxidant factors and prooxidant proteins are respectively changing, regulated by overall metabolism rate [72, 73]. For example MnSOD level is repeatedly changing from high in G1 and S phases to low in G2/M. The same was observed for its conformation and electrostatic potential [74]. To fulfill the increased demand for energy, cells in the G0/G1 phase increase the activity of proteins such as MnSOD, which are responsible for redox homeostasis. As was suggested by Sarsour *et al.*, eustress and oxidative stress are both crucial factors in cell cycle progression [72]. In response to oxidative damage or radiation stress MnSOD may be regulated through phosphorylation by cdc2 [75, 76]. Interestingly compounds 1b, 3b and 3c with strongest decreasing effect on cdc2 (Figure 6) caused also a large drop in the MnSOD level after 24 h of incubation (Figure 5). On the other hand however 2b and 3a increased the expression of cdc2. The cdc2 protein is involved in the transition from the G2 to M phase and its activity is sustained from the prophase to the metaphase during mitosis. Moreover, for 2b and 3a a relatively smaller increase in S phase and the highest increase in G2/M may be observed. Therefore, our results confirm cell cycle arrest in the S or G2 phase and the initiation of a mitotic catastrophe in HCT116 cells [77, 78]. On the other hand, Topham *et al.* postulated that the prolonged arrest of cells in the mitotic phase leads to their death *via* apoptosis, which is associated with the mitochondria signaling pathway [79].

Another possible mechanism of action, which characterizes most planar aromatic compounds, is intercalation of DNA. This can also be associated with interactions with transition metals such as iron, copper and nickel, which can form complexes with quinolines, 8-hydroxyquinolines and thiosemicarbazone derivatives [80–83]. Additionally, in previous studies, we indicated that the antiproliferative activity of quinoline-related compounds may be associated with the ability to intercalate into DNA, leading to the p53-independent mechanism of action of these compounds [49, 84]. Moreover, many iron chelators have been reported to be topoisomerase inhibitors or poisons [85]. The ability of ligands or metal complexes to bind to DNA may induce conformation changes, which as a result leads to DNA strand stress and damage [86]. In general interactions ligand-DNA may induce hypo- or hyperchromism. First is attributed with interaction of electronic state of compounds and those of DNA bases, increasing tightness of complex and π-stacking. The second effect, may reflect electrostatic binding (groove binders) or partial uncoiling of helix, as single stranded DNA revealed much stronger absorption as double helix. Strong hypochromism combined with red- or blue-shift is regarded as typical sign of intercalation. The stronger the effect and wavelength shift associated, the stronger interaction is. Our results shown in general high intercalating potency of TSC that were tested. An exception is control compound Dp44mT, for which only small effect was observed. This simple, dimethyl-substituted molecule is known for weak interactions with CT-DNA [48]. Thus our results indicate the importance of both of the substructures that are connected with the thiosemicarbazone moiety. Di-pyridine or quinoline rings and large aromatic rings instead of dimethylamine (compare 3a-c and Dp44mT), particularly the piperazine ring, may interact with minor groove DNA and thereby improve intercalation [87]. For this series of TSC strongest hypochromism and strong red- or blue-shift was observed. For substituted pyridine 3b the strongest hypopchromic effect was observed comparable with this of doxorubicin. On the other hand, series 2 of the thiosemicarbazones with the 8-hydroxyquinoline moiety had moderate intercalating properties. The combination of DNA intercalation and redox-active complexes may be responsible for the small amount of damage in the structure of the nucleic acids, which leads to cell cycle arrest as was indicated above. This hypothesis may explain the conflicting literature data suggesting that TSC do [88] or do not [89] interact directly with topoisomerases. This also corresponds with the fact that even non-intercalating TSC may induce some DNA damage via Cu-complexes [90].

To sum up the ionophoric nature of these chelators and a high activity in the Haber-Weiss and Fenton reactions are responsible for their complex interactions with the cellular antioxidant system. The aromatic structure and chelation ability of novel TSC is a prerequisite for DNA intercalation. Increasing the ROS levels beyond the restoration capacity of a cell affects the regulation of the MnSOD and CAT genes. This in turn up-regulates the Ndrg1 gene and leads to apoptosis. Despite the strong connection between iron metabolism and p53, all of the tested compounds triggered cell death in a p53-independent manner. These findings will hopefully help to understand and clarify the reports that are often contradictory.

## MATERIALS AND METHODS

### Synthesis

General reagents were purchased from Sigma-Aldrich (St. Louis, MO, USA) or ACROS Organics (Belgium). Thin layer chromatography (TLC) was performed on alumina-backed silica gel 40 F254 plates (Merck) and illuminated under UV (254 nm) The melting points were determined on Optimelt MPA100 instrument (SRS, USA) and are uncorrected. Syntheses were performed on a CEM-DISCOVERY microwave reactor (CEM Corporation, Matthews, NC, USA) with temperature and pressure control. High resolution mass spectrometry (HRMS) analysis was performed for all new compounds on SYNAPT G2-S HDMS (Waters, USA). The purity of all compounds was assessed using a Agilent1260 equipped with a DAAD detector at 260 nm, RP-column: Eclipse plus C18 (3,5 μm); flow 0.5 ml/min. The time of each measurement was 21 min. Conditions: 0− 0.8 min (80% H_2_O (0.1% TFA); 20% acetonitrile); 0.8−7 min (100% acetonitrile); 7−13 min (80% H_2_O (0.1% TFA); 20% acetonitrile).

All ^1^H NMR spectra were recorded on a Bruker AM-400 spectrometer (400 MHz) as well as Bruker AVANCE III (500 MHz). Chemical shifts are reported in ppm against the internal standard, Si(CH_3_)_4_. Easily exchangeable signals were omitted when diffuse. In general, thiosemicarbazones – 1a, 2a-b, 3a-c, Dp44mT and their thiosemicarbazide precursors were synthesized and characterized, as described previously [35, 36]. Doxorubicin was purchased from Sigma-Aldrich.

#### General procedure for the synthesis of thiosemicarbazones 1b-1c

The mixtures of (1,1′-thiocarbonyl) bis-1*H*-imidazole (5 mmol) and 4-(4-fluorophenyl)piperazine (5 mmol) or 4-[3-chloro-5-(trifluoromethyl)pyridin-2-yl]piperazine (5 mmol) in methylene chloride (25 mL) were stirred for 24 h at room temperature. The solutions were extracted three times with distilled water and the organic phases were dried over MgSO_4_ and evaporated. The obtained thioketones were refluxed for 2 hours with hydrazine hydrate (5 mmol). Finally, crude thiosemicarbazides were crystallized from dry methanol to obtain pure crystals. NMR spectra and mass spectrometry analysis is provided in Supplementary Figures 1–8.

4-(4-fluorophenyl)piperazine-1-carbothiohydrazide Light pink powder; yield 97%; mp:180–181° C; ^1^H-NMR (400 MHz, *d*_*6*_-DMSO, ppm): δ 3.09 (m, 4H, CH_2_), 3.87 (m, 4H, CH_2_), 4.77 (s, 2H, NH_2_), 6.97 (m, 2H, CH), 7.05 (m, 2H, CH), 9.19 (s, 1H, NH). ^13^C-NMR (101 MHz, *d*_*6*_-DMSO, ppm): δ 40.3; 49.1; 115.9; 117.9; 148.0; 157.8; 183.0.

4-[3-chloro-5-(trifluoromethyl)pyridin-2-yl]piperazine-1-carbothiohydrazide White powder; yield 85%; mp:191–192° C; ^1^H-NMR (500 MHz, *d*_*6*_-DMSO, ppm): δ 3.51 (m, 4H, CH_2_), 3.88 (m, 4H, CH_2_), 4.83 (s, 2H, NH_2_), 8.20 (d, 1H; *J* = 2.1 Hz), 8.56 (s, 1H, CH), 9.18 (s, 1H, NH). ^13^C-NMR (126 MHz, *d*_*6*_-DMSO, ppm): δ 47.3; 48.1; 120.2; 122.8; 125.0; 136.8; 143.5; 159.7; 183.2.

#### Microwave-assisted synthesis of thiosemicarbazones

Two drops of glacial acetic acid were added as a catalyst to the mixtures of thiosemicarbazides (0.5 mmol) and appropriate quinolinecarboxaldehyde (0.5 mmol) in ethanol (5 mL). The glass tubes were sealed and placed into a microwave reactor at 83° C for 20 minutes (the reactor power did not exceed 50 W). The final products were crystallized from methanol.

4-(4-fluorophenyl)-*N*’-[(quinolin-2-yl)methylidene]piperazine-1-carbothiohydrazide (1b) Yellow powder; yield 62%; mp:192–193° C; ^1^H-NMR (500 MHz, *d*_*6*_-DMSO, ppm): δ 3.26 (m, 4H, CH_2_); 4.13 (m, 4H, CH_2_); 7.02 (m, 2H, Ar-H); 7.09 (t, 2H, *J* = 8.9 Hz); 7.63 (m, 1H, Ar-H); 7.78 (m, 1H, Ar-H); 7.99 (m, 1H, Ar-H); 8.03 (m, 2H, Ar-H); 8.35 (s, 1H, CH); 8.39 (d, 1H, *J* = 8.7 Hz); 11.67 (s, 1H, NH). ^13^C-NMR (126 MHz, *d*_*6*_-DMSO, ppm): δ 49.4; 50.4; 115.8; 115.9; 117.8; 127.6; 128.2; 128.5; 129.3; 130.5; 137.2; 144.4; 147.9; 154.3; 155.8; 157.6; 181.2. HRMS-ESI: calcd for C_22_H_21_FN_4_S 394.1502 [M+H]^+^ found 394.1494.

4-[3-chloro-5-(trifluoromethyl)pyridin-2-yl]-*N*’-[quinolin-2-yl)methylidene]piperazine-1-carbothiohydrazide (1c) Yellow powder; yield 98%; mp:126–127° C; ^1^H-NMR (500 MHz, *d*_*6*_-DMSO, ppm): δ 3.69 (m, 4H, CH_2_); 4.14 (m, 4H, CH_2_); 7.62 (m, 1H, Ar-H); 7.79 (m, 1H, Ar-H); 8.01 (m, 3H, Ar-H); 8.23 (d, 1H, *J* = 2.2 Hz); 8.34 (s, 1H, CH); 8.38 (d, 1H, *J* = 8.7 Hz); 8.59 (dd,1H, *J* = 2.1, 0.9 Hz); 11.68 (s, 1H, NH). ^13^C-NMR (126 MHz, *d*_*6*_-DMSO, ppm): δ 19.0; 48.3; 50.2; 56.5; 117.8; 120.0; 127.6; 128.2; 128.5; 129.3; 130.5; 136.9; 137.1; 143.5; 144.4; 147.9; 154.3; 159.6; 181.6. HRMS-ESI: calcd for C_22_H_19_ClF_3_N_5_S 479.1033 [M+H]^+^ found 479.1025.

### Cell culture

The human colon cancer cell line HCT116 wild type and human breast carcinoma cell line MCF-7 were obtained from ATCC and the normal human fibroblast cell lines NHDF were obtained from PromoCell. Cells were grown as monolayer cultures in Dulbecco’s modified Eagle’s medium with an antibiotic gentamicin (200 μL/100 mL medium) in 75 cm^2^ flasks (Nunc). DMEM for HCT116 and MCF-7 were supplemented with 12% heat-inactivated fetal bovine serum (Sigma) and for NHDF with 15% non-inactivated fetal bovine serum (Sigma). Cells were cultured under standard conditions at 37° C in a humidified atmosphere at 5% CO_2_.

### Cytotoxicity studies

The cells were seeded in 96-well plates (Nunc) at a density of 3,500 cells/well (HCT116 and MCF-7) and 3,000 cells/well (NHDF) and incubated at 37° C for 24 h. The assay was performed following a 96 h incubation with varying concentrations of the compounds that were being tested. Then, 20 µL of CellTiter 96^®^ AQ_ueous_ One Solution-MTS (Promega) was added to each well (with 100 µL DMEM without phenol red) and incubated for 1 h at 37° C. The optical densities of the samples were analyzed at 490 nm using a multi-plate reader (Synergy 4, Bio Tek). Results were expressed as a percentage of the control and calculated as the inhibitory concentration (IC_50_) values (using GraphPad Prism 5). The IC_50_ parameter was defined as the compound concentration that was necessary to reduce the proliferation of cells to 50% of the untreated control. Each individual compound was tested in triplicate in a single experiment with each experiment being repeated three or four times.

### Impact of metal ions on cellular proliferation

The HCT116 and MCF-7 cells were seeded in 96-well plates (Nunc) at a density of 3,500 cells/well and incubated at 37° C for 24 h. The MTS assay was performed following a 96 h incubation with varying concentrations of the tested compounds. Additionally, a 20 µM solution of CuSO_4_, FeSO_4_ or FeCl_3_ was added into wells with the tested compounds. The results were expressed as a percentage of the control and calculated as the inhibitory concentration (IC_50_) values using GraphPad Prism 5. Each individual compound was tested in triplicate in a single experiment with each experiment being repeated three times.

### Reactive oxygen species formation – microscope images

The HCT116 cells were seeded into eight-well chambers (Lab-Tek) at a density of 0.5 × 10^5^ cells/well and incubated at 37° C. After 24 h, freshly prepared solutions of the tested compounds: 1b, 1c, 2a-b, 3a-c and DOX (two-fold IC_50_ concentration) were added. The next day, the solutions of the tested compounds were removed and the cells were washed with Phosphate Buffered Saline (PBS), after which 5 μM CellROX^®^ Green Reagent (Molecular Probes^™^) was added. After 30 min of incubation at 37° C, the cells were washed with PBS and then DMEM without phenol red was added. The observation was performed using an inverted fluorescence microscope (IX81, Olympus) equipped with a CO_2_ incubator using a 485 nm excitation laser and a 520 nm emission filter.

### Time dependent measurement of ROS level

To determine the intracellular levels of ROS, HCT116 cells were seeded onto black 96-well plates (Corning) at a density of 9,000 cells/well and incubated at 37° C. After an overnight incubation, the solutions of the tested compounds: 1a-c, 2 a-b, 3 a-c, Dp44mT (1 μM) and DOX (5 μM) were added and incubated for 3, 6, 9, 12, and 24 h in a kinetic experiment. The generation of ROS was measured using a CellROX^®^ Green Reagent (Molecular Probes™). Additionally, the quantity of cells in each well was determined using Hoechst 33342 (Molecular Probes™). The solutions of the tested compounds were removed and 100 µL of CellROX Green Reagent and Hoechst 33342 at a final concentration of 5 μM were added to each well. Then, the cells were incubated for 30 min at 37° C. The fluorescence was measured using a multi-plate reader (Synergy 4, Bio Tek) at 485 nm excitation and a 520 nm emission for CellROX Green Reagent and a 345 nm excitation laser and a 485 nm emission filter for Hoechst 33342. The experiments were performed three to four times. ROS levels were expressed as the percentage of the control cells level.

### Time dependent measurement of glutathione level

To determine the intracellular level of reduced glutathione, the HCT116 cells were seeded in 3 cm Petri dishes (Nunc) at a density of 0.5 × 10^6^ cells/well and incubated at 37° C. The next day, solutions of TSC (1 μM) and DOX (5 μM) were added and incubated for 3, 6, 9, 12, and 24 h in a kinetic experiment. The levels of intracellular GSH were measured using an enzymatic recycling method [91]. Briefly, 20 μL of each cell lysate was transferred into 96-well plates. Then, freshly prepared solutions of 5,5′-dithio-bis(2-nitrobenzoic acid) (DNTB) at a final concentration of 0.67 mg/mL and glutathione reductase (GR) – 1.67 units/mL, were added to each well. After 30 sec, β-NADPH (0.67 mg/mL) was added to each well and the absorbance was immediately measured at 412 nm in a multi-plate reader (Synergy 4, Bio Tek). The experiments were performed in triplicate. Data were expressed as the percentage of the control cells level.

### Analysis of the mRNA expression of MnSOD, CAT, Ndrg1

The HCT116 cells were seeded in 3 cm Petri dishes (Nunc) at a density of 0.5·10^6^ cells/well and incubated overnight. Next, the medium was removed and solutions of TSC (1 μM) and DOX (5 μM) were added. After 12 or 24 h, total RNA was isolated from the cells using TRIzol Reagent (Ambion) according to the manufacturer’s instructions. cDNA synthesis was performed with 5 μg of total RNA using a GoScript^™^ Reverse Transcriptase kit (Promega) and Oligo(dT)_23_ Primers (Sigma). The Real-Time PCR was performed with a CTX96 Touch^™^ Real-Time PCR Detection System (Biorad) in a 20 μL reaction volume. The reaction consisted of PowerUp^™^ SYBR^®^ Green Master Mix (Applied Biosystems), a primer pair mix (0.5 μM each) and 1 μL of cDNA. All primer pair sequences were purchased from Sigma-Aldrich and are listed in Supplementary Table 2. The experiment was performed under the following conditions: initial denaturation at 95° C for 120 sec; followed by 40 denaturation cycles at 95° C, 15 sec; annealing (primer-specific temperature for 30 sec) and extension at 72° C for 60 sec. Data was analyzed based on a comparison of the expression of the target genes to a reference gene – β-actin, using the 2^–ΔΔCT^ method. The experiments were performed at least three times.

### Immunoblotting

The HCT116 cells were seeded in 3 cm Petri dishes (Nunc) at a density of 0.5·10^6^ cells/well and incubated overnight. The next day, solutions of TSC (1 μM) and DOX (5 μM) were added and the cells incubated for 24 or 48 h. Cells were harvested by trypsinization and washed with cold PBS. Next, the cells were centrifuged and suspended in an RIPA buffer (Thermo Scientific) containing Halt Protease Inhibitor Cocktail (Thermo Scientific), Halt Phosphatase Inhibitor Cocktail (Thermo Scientific) along with 0.5 M EDTA and lysed for 20 min on ice. Then, the lysates were sonicated, centrifuged at 10,000 rpm for 10 min at 4° C and the supernatants were collected for further analysis. The protein concentration was determined using a Micro BCA^™^ Protein Assay Kit (Thermo Scientific) according to the manufacturer’s instructions. Equal amounts of the proteins (20 μg) were electrophoresed on SDS-Page gels and transferred onto a nitrocellulose membranes. The membranes were blocked in 5% non-fat milk prepared in PBS containing 0.1% Tween-20 (TPBS) for 1 h. After blocking, the membranes were incubated with specific primary antibodies: cdc2 (#POH1, Cell Signaling), p21^Waf1/Cip1^ (#12D1, Cell Signaling), p53 (#1C12, Cell Signaling) and β-Actin (#8H10D10, Cell Signaling) overnight at 4° C, then washed and incubated with horseradish peroxidase (HRP)-conjugated secondary antibodies for 1h at room temperature. All of antibodies were diluted 1:1000 in 5% milk in TPBS. Finally, the membranes were washed and incubated with a SuperSignal^™^ West Pico Chemiluminescent Substrate (Thermo Scientific). The chemiluminescence signals were captured using a ChemiDoc^™^ XRS+ System (BioRad). The experiments were performed at least three times. Densitometric analysis was performed using ImageJ 1.41 software (Wayne Rasband, National Institutes of Health, USA).

### Cell cycle assay

The HCT116 cells were seeded in 3 cm Petri dishes (Nunc) at a density of 0.25·10^6^ cells/well and incubated at 37° C for 24 h. Then, the medium was removed and freshly prepared solutions of the tested TSC (0.5 μM) and DOX (5 μM) were added. After a 48 h treatment, assays were performed using a Muse Cell-Cycle Kit (Millipore) according to the manufacturer’s instructions. Briefly, cells were collected, washed with cold PBS and centrifuged at 300 g. Then, the cells were fixed in ice cold 70% ethanol and stored at –20° C overnight. Afterwards, the cells were centrifuged and resuspended in 200 μL of Muse^™^ Cell Cycle Reagent and incubated for 30 min at room temperature in the dark. After staining, the cells were processed for cell cycle analysis using a Muse Cell Analyzer (Millipore). The experiments were performed at least three times.

### Annexin V binding assay

The HCT116 cells were seeded in 3 cm Petri dishes (Nunc) at a density of 0.25·10^6^ cells/well and incubated at 37° C for 24 h. Then, the medium was removed and freshly prepared solutions of the tested TSC (0.5 μM) and DOX (5 μM) were added. After 48 h, assays were performed using an Annexin V & Dead Cell Kit (Millipore) according to the manufacturer’s instructions. Briefly, detached and adherent cells were collected and centrifuged at 500 g for 5 min. Afterwards, the resuspended cells were incubated with 100 μL of Muse^™^ Annexin V & Dead Cell Reagent for 20 min at room temperature in the dark. After staining, the events for live, early and late apoptotic cells were counted using a Muse Cell Analyzer (Millipore). The experiments were performed at least three times.

### Intercalation

For the DNA binding studies, Calf-thymus DNA (CT-DNA) was purchased from Sigma Aldrich. The lyophilized CT-DNA was dissolved in 10 mM Tris-HCl, pH 7.9, mixed gently and left overnight at 4° C. The purity of the CT-DNA solution was determined by measuring the ratio of UV absorbance at 260 and 280 nm. A ratio more than 1.8 indicated that the DNA was sufficiently free from proteins. Then, the concentration of CT-DNA was determined from the absorbance at 260 nm using an extinction coefficient of 6600 M^–1^cm^–1^. The tested TSC and DOX were dissolved in DMSO to a concentration of 8.35 mM, which was then used as the stock solution for preparing the various concentrations (25, 12.5, 6 and 3 µM) in 1 mL in 10 mM of Tris-HCl (pH 7.9). Afterwards, 18 µM CT-DNA was added to the prepared solutions, which were incubated for 1.5 h at 37° C with occasional vortexing. The absorption spectra were measured using a Hitachi U-2900 spectrophotometer in range of 200–500 nm. All absorption spectra were imported and compared in OriginPro 8.0.

### Statistical analysis

Results are expressed as the mean ± standard deviation (SD) from at least three independent experiments. Statistical analysis in the ROS, GSH measurements and expression of genes were performed using the two-tailed Student’s *t*-test. Statistical differences in the expression of proteins, progression of cell cycle and Annexin V binding assay were calculated using one-way ANOVA with a Bonferroni post-hoc test. A *p*-value of 0.05 or less was considered to be statistically significant. GraphPad Prism v.5.0 software (GraphPad Software, USA) was used for analysis.

## SUPPLEMENTARY MATERIALS FIGURES AND TABLES



## Abbreviations

ATP: adenosine triphosphate
CAT: catalase
CT-DNA: calf-thymus DNA
DOX: doxorubicin
GSH: glutathione
GSSG: oxidized glutathione
MDR: multi-drug resistance
MnSOD: manganese superoxide dismutase
Ndrg1: N-myc downstream regulated gene 1
PDT: photodynamic therapy
ROS: reactive oxygen species
RR: ribonucleotide reductase
TSC: thiosemicarbazones
