# Supplementary material for: The role of oxidative stress in activity of anticancer thiosemicarbazones
Source: Oncotarget. 2018 Apr 3;9(25):17689–710. doi: 10.18632/oncotarget.24844 (PMC5915149; doi:10.18632/oncotarget.24844)
Supplement: Supplementary file 1 [file oncotarget-09-17689-s001.pdf]

The role of oxidative stress in activity of anticancer thiosemicarbazones

SUPPLEMENTARY MATERIALS

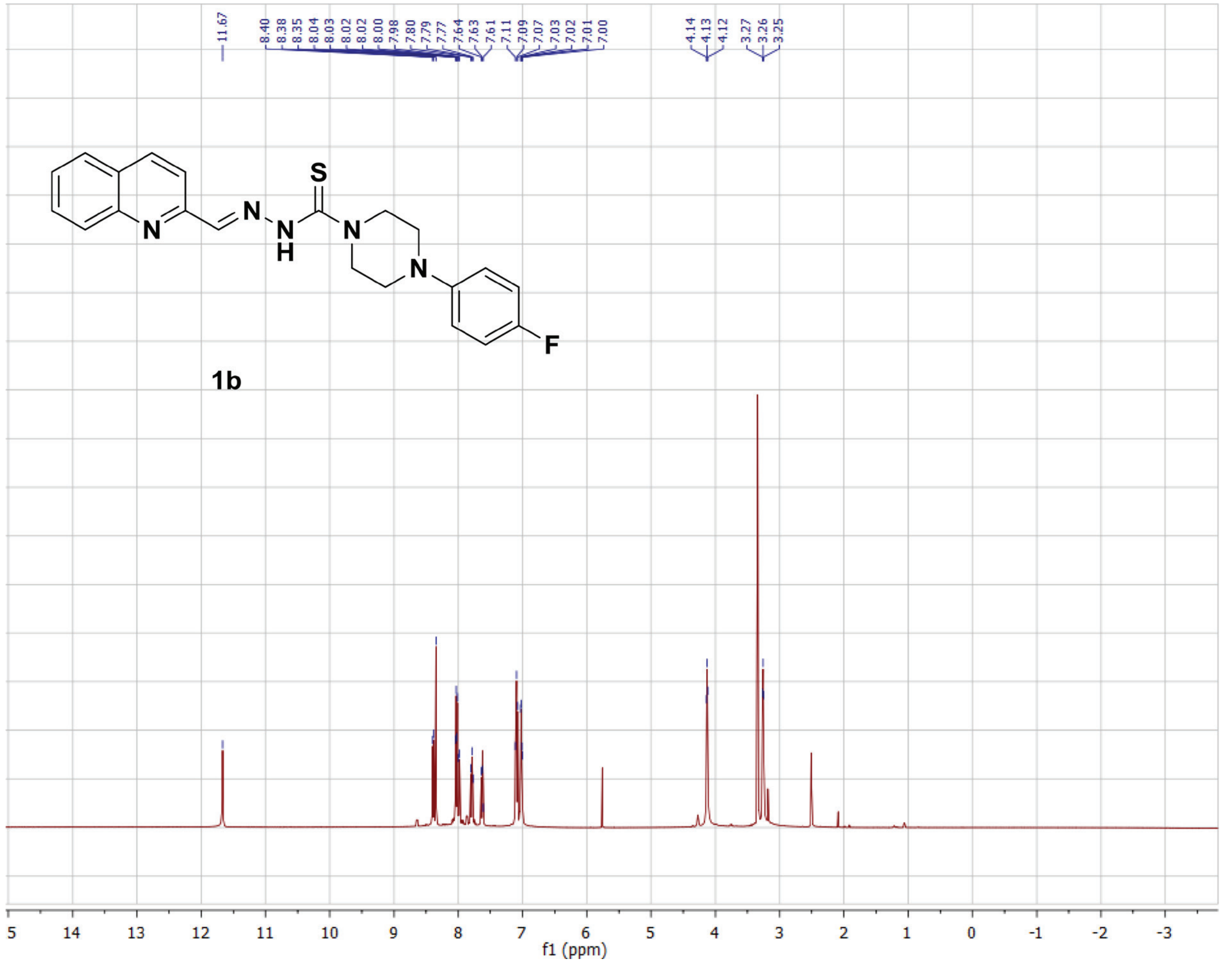

Supplementary Figure 1: ^{1}H-NMR spectrum of 1b in DMSO.

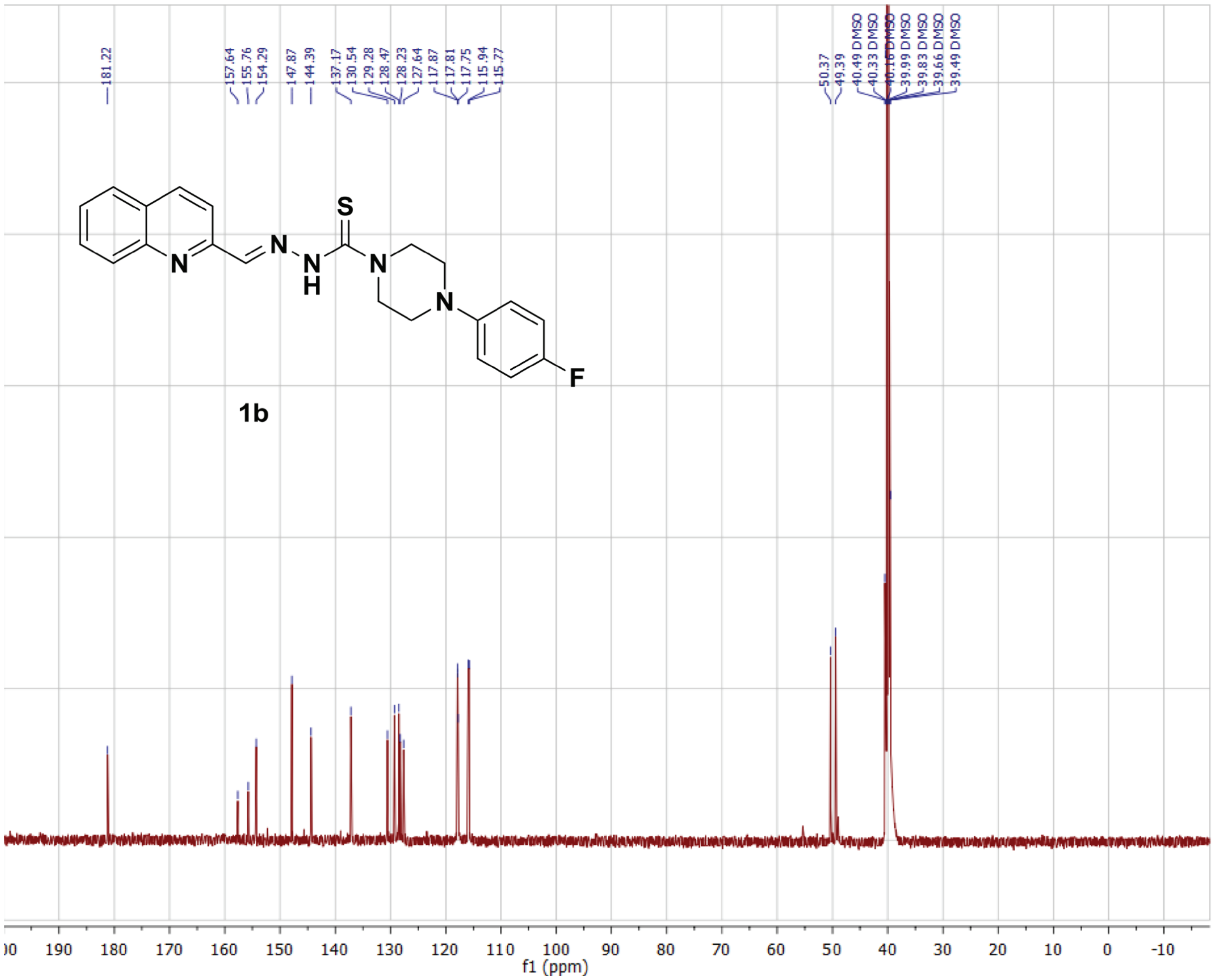

Supplementary Figure 2: ^{13}C-NMR spectrum of 1b in DMSO.

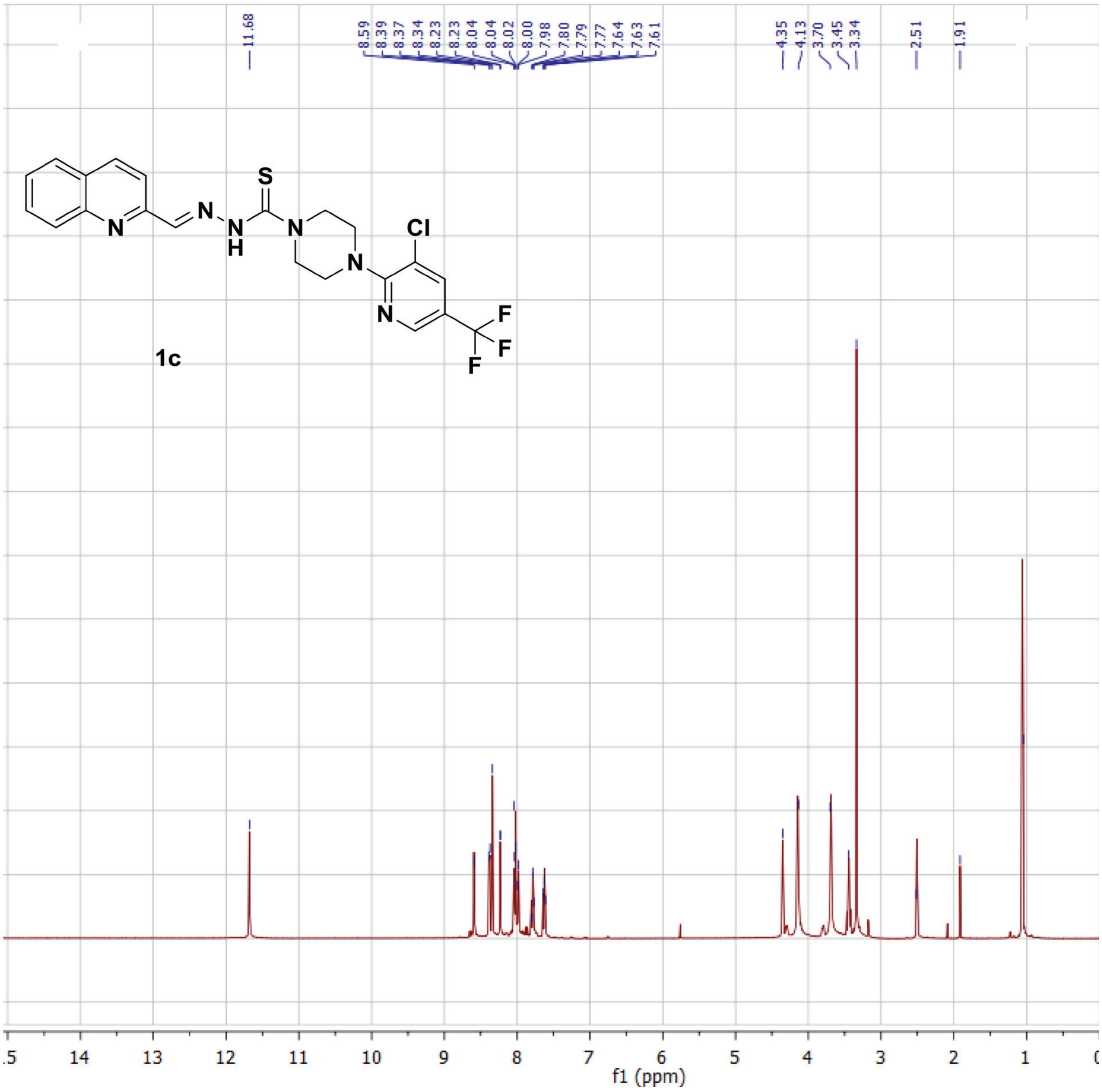

Supplementary Figure 3: ^{1}H-NMR spectrum of 1c in DMSO.

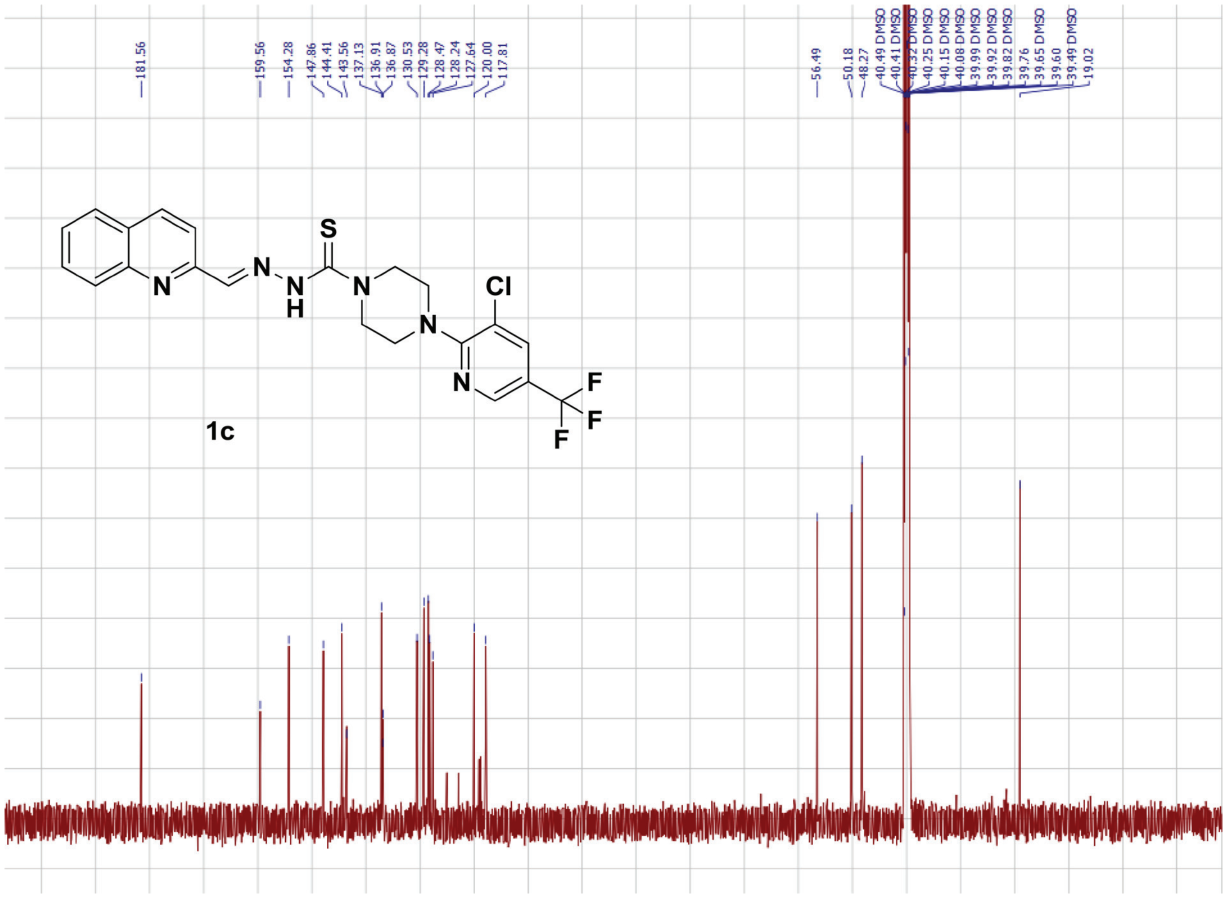

Supplementary Figure 4: ^{13}C-NMR spectrum of 1c in DMSO.

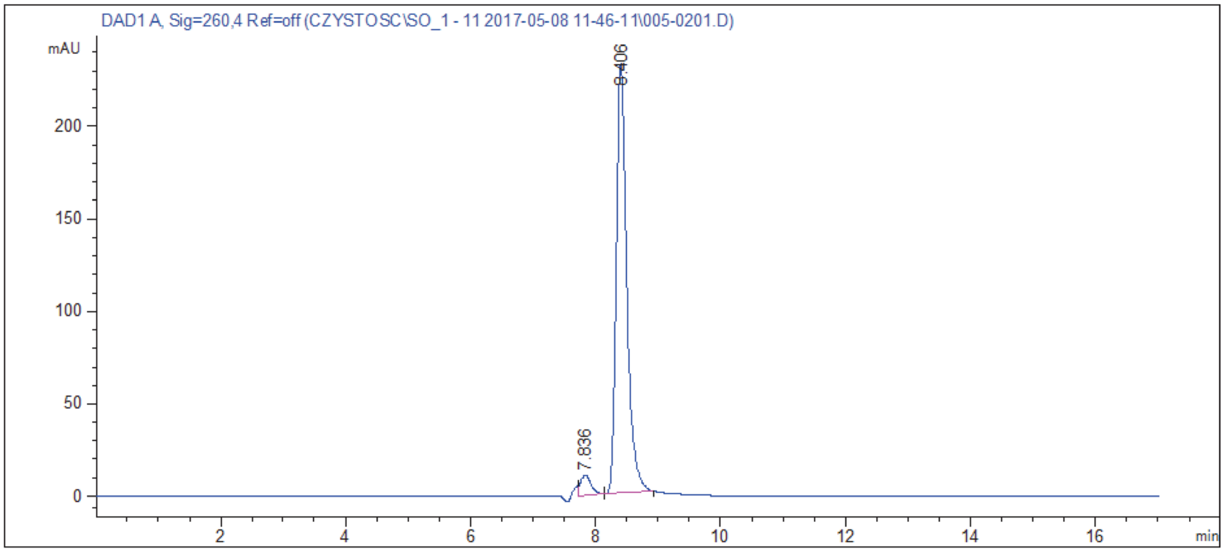


```
=====================================================================
                           Area Percent
                              Report
=====================================================================

Sorted By             :      Signal
Multiplier            :      1.0000
Dilution              :      1.0000
Do not use Multiplier & Dilution Factor with ISTDs


Signal 1: DAD1 A, Sig=260,4 Ref=off

Peak RetTime Type  Width     Area      Height     Area
  #   [min]        [min]   [mAU*s]     [mAU]        %
----|-------|----|-------|----------|----------|--------|
   1   7.836 FM    0.1893  124.46830   10.95627   4.3745
   2   8.406 FM    0.1950 2720.84155  232.51500  95.6255

Totals :                   2845.30985  243.47127


=====================================================================

=====================================================================
                           Summed Peaks
                              Report
=====================================================================
```

Supplementary Figure 5: RP-HPLC analysis of 1b purity.

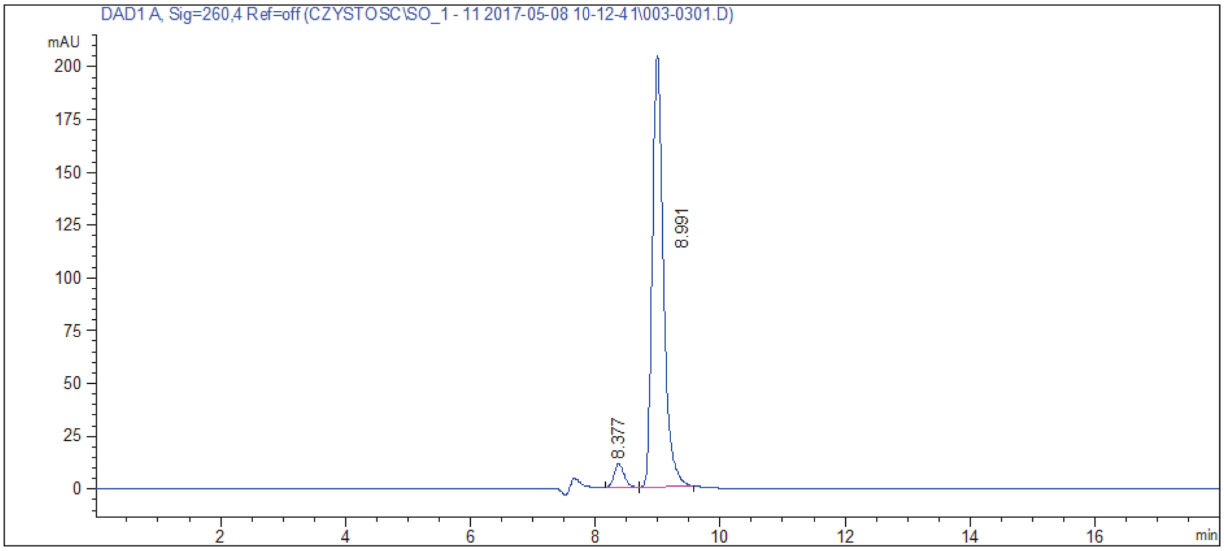

==

Area Percent Report

==

Sorted By : Signal
Multiplier : 1.0000
Dilution : 1.0000
Do not use Multiplier & Dilution Factor with ISTDs

Signal 1: DAD1 A, Sig=260,4 Ref=off

Peak #	RetTime [min]	Type	Width [min]	Area [mAU*s]	Height [mAU]	Area %
1	8.377	BB	0.1673	119.45763	11.12156	4.5408
2	8.991	MM	0.2049	2511.30981	204.27605	95.4592

Totals : 2630.76744 215.39761

==

==

Summed Peaks Report

==

Supplementary Figure 6: RP-HPLC analysis of 1c purity.

Elements Used:
C: 0-100 H: 0-100 N: 4-6 S: 1-1 F: 1-1

Mass	Calc. Mass	mDa	PPM	DBE	Formula	i-FIT	i-FIT Norm	Fit Conf %	C	H	N	S	F
394.1494	394.1502	-0.8	-2.0	13.5	C21 H21 N5 S F	839.9	n/a	n/a	21	21	5	1	1

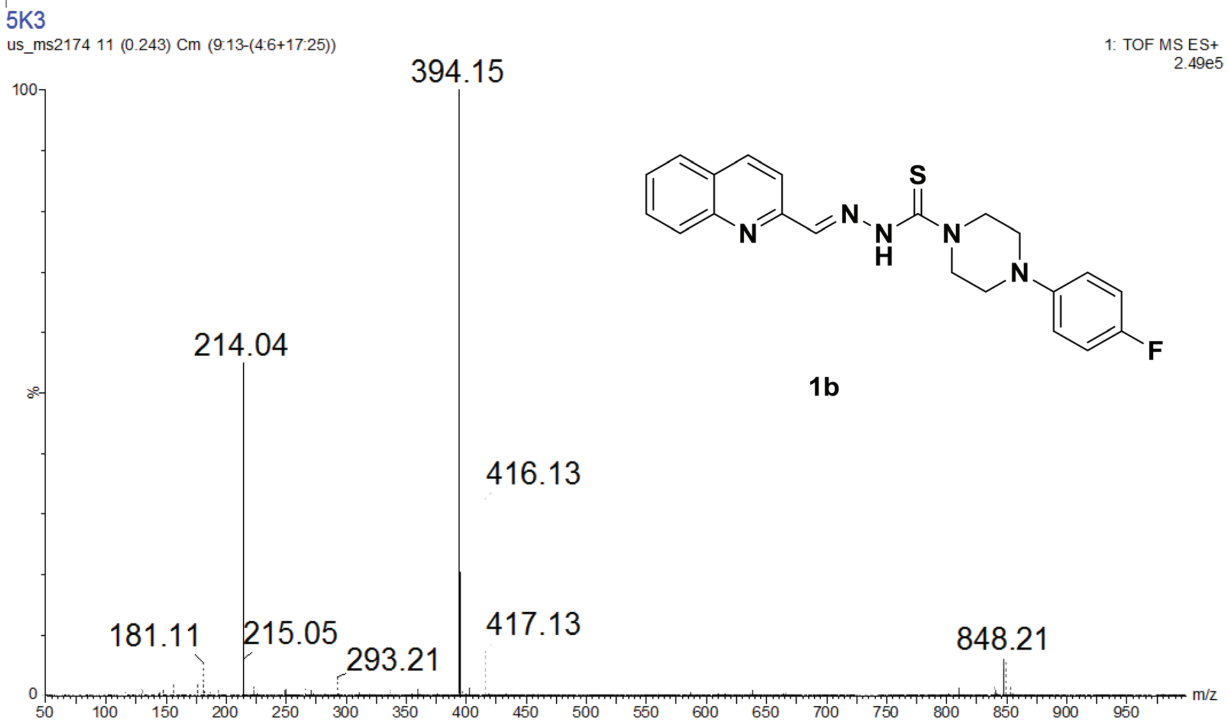

Supplementary Figure 7: HR-ESI spectrum of 1b.

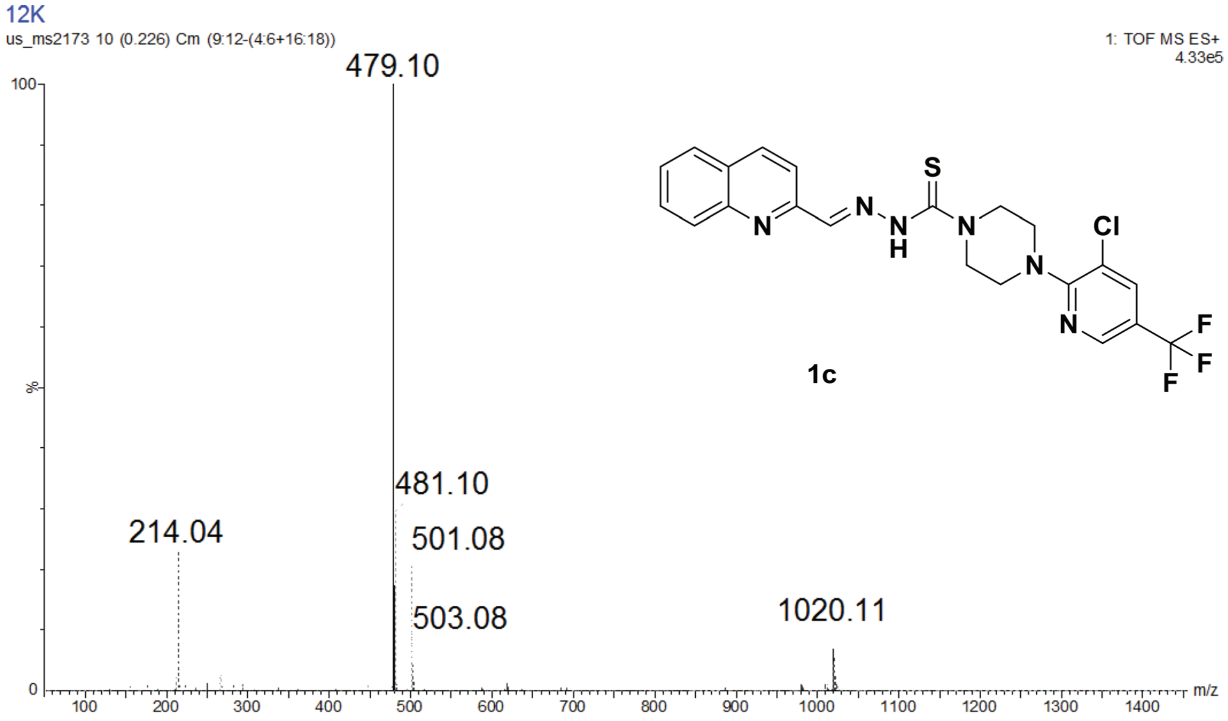

Elements Used:
C: 0-50 H: 0-50 N: 5-7 S: 1-1 Cl: 1-1 F: 3-3

Mass	Calc. Mass	mDa	PPM	DBE	Formula	i-FIT	i-FIT Norm	Fit Conf %	C	H	N	S	Cl	F
479.1025	479.1033	-0.8	-1.7	13.5	C21 H19 N6 S Cl F3	774.7	n/a	n/a	21	19	6	1	1	3

Supplementary Figure 8: HR-ESI spectrum of 1c.

Supplementary Table 1: Selectivity indexes of the studied compounds, calculated for HCT-116 and MCF-7 lines

Comp.	HCT116	MCF-7
1a	107.6	43.1
1b	1.8	3.6
1c	236.6	989.6
2a	2770.8	978.8
2b	240.4	376.7
3a	2.1	0.1
3b	77.7	39.0
3c	0.4	0.5
Dp44mT	10985.7	40473.7
DOX	1.5	0.3

Supplementary Table 2: Primer pair sequences used to determine the mRNA expression of MnSOD, CAT, Ndrg1 and β-actin

Gene	GenBank accession no.	Forward primer (5′→3′)	Reverse primer (5′→3′)
MnSOD	NM_001024465.1	AAACCTCAGCCCTAACGGTG	CCAGGCTTGATGCACATCTTA
CAT	NM_001752.3	ACTGTTGCTGGAGAATCGGG	AAGTCTCGCCGCATCTTCAA
Ndrg1	NM_006096.3	GTCTCGGGAGATGCAGGATG	TGTGGTTCATGCCGATGTCA
β-actin	NM_001101.3	CTCGCCTTTGCCGATCC	GCTGGGGTGTTGAAGGTCTC
